# Cell death pathways in response to *Mycobacterium tuberculosis* and other mycobacterial infections

**DOI:** 10.1128/iai.00401-25

**Published:** 2025-09-09

**Authors:** Md Atik Faysal, Mostafa Hanafy, Denise K. Zinniel, Fatema Yeasmin Tanni, Ezhumalai Muthukrishnan, Govardhan Rathnaiah, Raul G. Barletta

**Affiliations:** 1 School of Veterinary Medicine and Biomedical Sciences, University of Nebraska 684783, Lincoln, Nebraska, USA; 2 Department of Microbiology and Immunology, Faculty of Veterinary Medicine, Cairo University 540151, Giza, Egypt; 3 Department of Biochemistry, University of Nebraska 315569, Lincoln, Nebraska, USA; 4 Eppley Institute for Research in Cancer and Allied Diseases, University of Nebraska Medical Center 272558, Omaha, Nebraska, USA; University of California Davis, Davis, California, USA

**Keywords:** *Mycobacterium*, macrophages, cell death, autophagy, apoptosis, necrosis, ferroptosis, pyroptosis

## Abstract

Cell death mechanisms play a fundamental role in mycobacterial pathogenesis. We critically reviewed 94 research manuscripts, 44 review articles, and 4 book chapters to analyze important discoveries, background literature, and potential shortcomings in the field. The focus of this review is the pathogen *Mycobacterium tuberculosis* (Mtb) and other Mtb and *Mycobacterium avium* complex microorganisms. Virulent strains hijack cell death processes by inhibiting autophagy, apoptosis, and pyroptosis while eliciting necrosis and ferroptosis to multiply intracellularly and spread within and between hosts. In addition, virulent strains may induce apoptosis in epithelial cells or secondary infected macrophages to spread. Autophagy does not control Mtb intracellular replication *in vivo* but suppresses macrophage and T cell responses in Mtb infections, with a predominant role in preventing neutrophil infiltration. In contrast, attenuated vaccine strains promote apoptosis in macrophages, leading to the activation of innate immunity and, eventually, the acquired immune response. Although Mtb infection activates necroptosis, studies with mutant cell lines have indicated that this process is not essential for cell lysis and that Mtb promotes unprogrammed necrosis. Ferroptosis is discussed in the context of necrotic processes involving lipid peroxidation. Recent research indicated that pyroptosis is more akin to apoptosis as Mtb proteins induce cell membrane repair to prevent inflammasome activation. In the supplementary tables, homologs of mycobacterial cell death pathways and virulence factors were identified using a basic local alignment search tool protein followed by a conserved domain database search to determine the presence of functional domains. Finally, prospects for therapeutic interventions are discussed.

## INTRODUCTION

Mycobacteria are intracellular pathogens causing chronic inflammatory diseases in humans and animals. Tuberculosis (TB) is a major public health concern with *Mycobacterium tuberculosis* (Mtb) as the etiologic agent. Based on the World Health Organization 2024 Global Tuberculosis Report, TB accounted for 8.2 million newly diagnosed cases and an estimated 1.25 million deaths in 2023, positioning this disease as the leading cause of mortality due to a singular infectious agent (1). Another species of clinical significance is *Mycobacterium tuberculosis* variant *bovis* (Mbo), the agent of zoonotic TB that is transmitted from/to humans, wildlife, and domestic animals (2). Other important pathogens include *Mycobacterium avium* subsp. *hominissuis* that infects immunocompromised human hosts and swine (3, 4), and *Mycobacterium avium* subsp. *paratuberculosis* (Map), which is the agent of Johne’s disease in ruminants and has been associated with Crohn’s disease in humans (5). This review focuses on how mycobacteria interact with macrophages, neutrophils, and other immune cells to upregulate or downregulate autophagy and cell death processes of apoptosis, necrosis, necroptosis, ferroptosis, and pyroptosis. We discuss the implications for mycobacterial virulence and candidate live-attenuated vaccines as evidenced from animal models and natural hosts, as well as disease progression outcomes.

## CELL DEATH AND MYCOBACTERIAL INFECTIONS

Cell death is a fundamental aspect of host homeostasis and innate immunity to microbial infections. These processes benefit the host by eradicating pathogens at initial stages by stimulating dendritic cells (DC) to engulf deceased host cells and pathogens. Subsequently, these DC present antigens on their surfaces, leading to the onset of acquired immunity. Interactions with macrophages, natural killer cells, and neutrophils involve prompting innate immune cells to secrete cytokines, subsequently triggering the acquired immune response (IR) (6). Conversely, some cell death mechanisms favor the propagation of pathogens. Intracellular bacterial pathogens employ diverse strategies to multiply within phagocytic cells, manipulate cell death pathways, and elude immune detection (7). Pathogens manipulate these pathways utilizing diverse tactics, contingent upon both the bacterial species and the type of host cell.

Macrophages are pivotal in the fight against TB and other mycobacterial diseases (5, 6, 8). Mtb enters the host through aerosols, with subsequent interactions with pathogen recognition receptors within phagocytic cells through its pathogen-associated molecular patterns. Mtb has evolved strategies to outmaneuver host defenses and establish infection by inducing intracellular signaling that activates inflammatory cytokine/chemokine responses to eventually downregulate the protective IR. These mechanisms are instrumental in the development of immunopathological reactions or thwarting Mtb replication for a favorable host outcome (9). Surface receptors involved are toll-like receptors (TLR), DC-specific intercellular adhesion molecule-3-grabbing nonintegrin, and mannose receptors. Intracellular receptors include the MyD88 adapter molecule, proteins with the nucleotide oligomerization domain (NOD), and the caspase activation and recruitment domain (CARD). These events activate a signaling cascade that culminates in the onset of transcription via the nuclear factor-kappa B (NF-κB) (10, 11). Mtb hampers processes like autophagy, apoptosis, phagosome-lysosome fusion, and antigen presentation. Moreover, Mtb inhibits the formation of reactive oxygen (ROS) and nitrogen (RNS) species from the host, thereby facilitating its survival within macrophages (12).

We will describe the interaction of Mtb with the phagosomal compartments as recently reviewed (13). Briefly, upon phagocytosis by resident alveolar macrophages, Mtb is contained in an early phagosome whose maturation into a late phagosome is promoted by RAB5, RAB7, and RAB7L1 proteins. However, Mtb virulence factors (e.g., PknG, Rv0410c; NdkA, Rv2445c; and SapM, Rv3310) attempt to suppress this process by inhibiting trafficking to phagolysosomes using bacterial lipid factors whose synthesis and/or transport require various proteins: (i) diacyltrehalose synthesized by Pks3 (Rv1180), Pks4 (Rv1181), PapA3 (Rv1182), and FadD21 (Rv1185c); (ii) penta-acyltrehalose transported or synthesized by MmpL10 (Rv1183) and Chp2 (Rv1184c); (iii) lipoarabinomannan and the corresponding proteins Rv1635c and Rv2181; (iv) phosphatidylinositol mannoside with proteins PimE (Rv1159), PimB (Rv2188c), and PimA (Rv2610c); (v) sulfoglycolipid 1 transport protein MmpL8 (Rv3823c); and biosynthetic proteins PapA2 (Rv3820c) and PapA1 (Rv3824c) are required as shown by deletion mutant studies (14); and (vi) trehalose dimycolate (TDM) and the cutinase/TDM hydrolase Cut3 (Rv3451) (15). Moreover, once Mtb is in this compartment, full acidification is prevented by the exclusion of the vacuolar-type ATPase mediated by the phosphotyrosine phosphatase PtpA (Rv2234). Indeed, interstitial and monocyte-derived macrophages are better able to control Mtb than alveolar macrophages.

### Mtb subverts xenophagy *in vitro,* but autophagy proteins control neutrophil infiltration *in vivo*

Autophagy involves cellular phagocytosis of damaged organelles, due to infection or other pathological processes, in which a cell recycles its damaged, outdated, and malfunctioning internal components to provide energy and support new and healthy cell growth (16). This process is usually characterized by the formation of autophagosomes with a double membrane surrounding organelles and part of the cytoplasmic matrix (17). Eventually, autophagosomes fuse with lysosomes, and the content is degraded within these autophagolysosomes. However, autophagy is not a cell death process but rather a survival mechanism to protect cells and control excessive immune activation. If inadequate, cells undergo death by apoptosis or necrosis/necroptosis.

**Fig 1:**
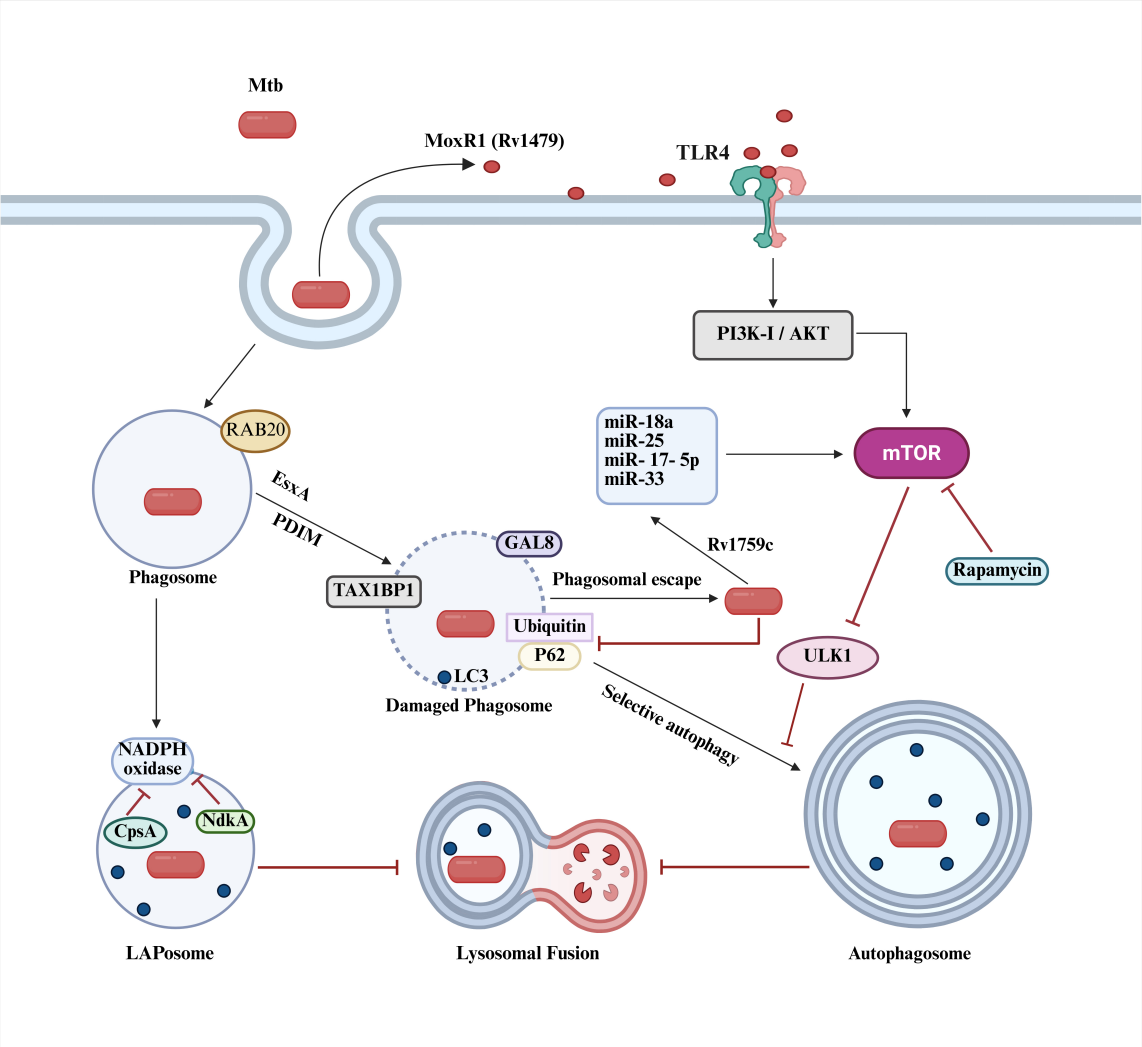
Inhibition of autophagy by Mtb in macrophages. The main Mtb and host components are displayed. The black pointed head arrows indicate activating processes while red blunt head arrows indicate inhibition. Generated by Biorender.com.

At the cellular level, virulent Mtb has various effector molecules that counter autophagy early in the macrophage uptake process. Mtb employs the secreted proteins, PE_PGRS20 (Rv1068c), PE_PGRS47 (Rv2741), and LprE (Rv1252c), to inhibit autophagy (18, 19). Another protein, MoxR1 (Rv1479), attaches to the host receptor TLR4, triggering the release of pro-inflammatory cytokines and the phosphoinositide 3-kinases-protein kinase B-mammalian target of rapamycin (PI3K-PKB [a.k.a. AKT]-mTOR) signaling cascade (Fig. 1). This leads to the upregulation of mTOR and the inactivating phosphorylation of UNC-51-like kinase 1 (ULK1) inhibiting autophagosome formation (20). In addition, microRNAs, such as miR-17-5p, miR-18a, miR-25 (induced by Rv1759c, Wag22), and miR-33, lead to the upregulation of mTOR and downregulation of ULK1 with the same final outcome of suppressing autophagy and promoting lipid body formation, which serve as nutrients. Macrophages can deliver Mtb to an alternative route via the RAB20 protein in an attempt to encapsulate the pathogen into a microtubule-associated protein 1 light chain 3 (LC3)-decorated autophagosome, leading to xenophagy (selective autophagy of invading pathogens) (13). During this process, the surface protein Rv1468c (PE_PGRS29) binds ubiquitin, while the secreted protein MPT53 (Rv2878c) promotes downstream signaling to produce inflammatory cytokines protecting the host (21, 22). In contrast, ubiquitination can also play a dual role via Rv0222 and PtpA that leads to the IR suppression (23–25).

Mtb counteracts autophagy with the type VII secretion system effector proteins EsxH (Rv0288) and EsxA (Rv3875, ESAT-6) in addition to the lipid phthiocerol dimycocerosate (PDIM) and the corresponding thioesterase (Rv2928, TesA) that cooperate to promote phagosomal membrane damage (26). The cytosolic glycan-binding protein galectin-8 (GAL8) encounters the xenophagy adapter Tax1-binding protein 1 (TAX1BP1) and helps to recognize the damaged phagosome containing Mtb. Overexpression of GAL8 enhances the ability of the macrophage to target Mtb to autophagy and restrict growth (27). The host protein ubiquilin 1 participates in this process by stimulating the production of interferon-gamma (IFN-γ) and the accumulation of ubiquitin, p62, and LC3 on Mtb leading to autophagy (28). In macrophages not activated by IFN-γ, Mtb inhibits this xenophagic process, eventually escaping into the cytosol and promoting necrosis. In addition, LC3 and NADPH oxidase bind to this phagosome, but several proteins (e.g., CpsA, Rv3484; KatG, Rv1908c; NdkA; NuoG, Rv3151; and PPE2, Rv0256c) impair the recruitment of the oxidase, preventing the formation of a mature LAPosome (LC3-associated phagocytosis Mtb phagasome) delivering Mtb to the phagolysosome. In addition, Eis (Rv2416c) inhibits autophagy to enhance intracellular survival in bone marrow-derived macrophages (BMDM) from C57BL/6 mice (29).

Research with C57BL/6 mice conditionally deleted in the autophagy-related 5 (*Atg5*) gene in CD11c+ cells infected with a low Mtb dose revealed a different perspective that undermines the significance of the cellular studies described above in this section (30). The role of *Atg5* in lung macrophages and DC (CD11c+ cells) is autophagy dependent and essential for preventing the influx of neutrophils into pulmonary tissue during infection. Increased neutrophils recruitment leads to a poor TB disease outcome. Moreover, the effect of *Atg5* is independent of other Mtb effector mechanisms like mitophagy (mitochondrial autophagy) or inflammasome activation. The importance of neutrophils in a low Mtb dose was confirmed in BMDM and mice, based on a different gene deletion methodology, indicating that autophagy is important in the defense against Mtb infections in mammals (31). Another study to simulate active TB used a high Mtb dose to infect mice, each with deletions of one autophagy gene (*Atg5, Atg7, Atg161l, Atg14, Fip200,* and *Becn1*) in CD11c+ cells (32). The loss of autophagy increased mice susceptibility, and it was concluded that autophagy does not control Mtb intracellular replication *in vivo* but suppresses macrophage responses due to the overproduction of myeloid-derived suppressor cells and subsequent impairment in T cell responses. These immature myeloid cells are not fully differentiated neutrophils, monocytes/macrophages, or DC but are derived from a common myeloid progenitor. Thus, autophagy in CD11c+ cells suppresses neutrophil recruitment during both low and high (more severe effect) Mtb dose. A recent study using mice lacking ATG5 in neutrophils showed that ATG5 also functions independently of autophagy to limit type I IFN-induced processes, especially the release of neutrophil extracellular traps and the secretion of the pro-inflammatory chemokine CXCL2 (33). Loss of ATG5-mediated suppression of these processes in neutrophils results in excessive neutrophil accumulation in the lungs, promoting severe inflammation and increased susceptibility to TB, a key finding underlying the human disease spectrum and potential therapies.

### Mtb prevents apoptosis in macrophages while attenuated strains do not

**Fig 2:**
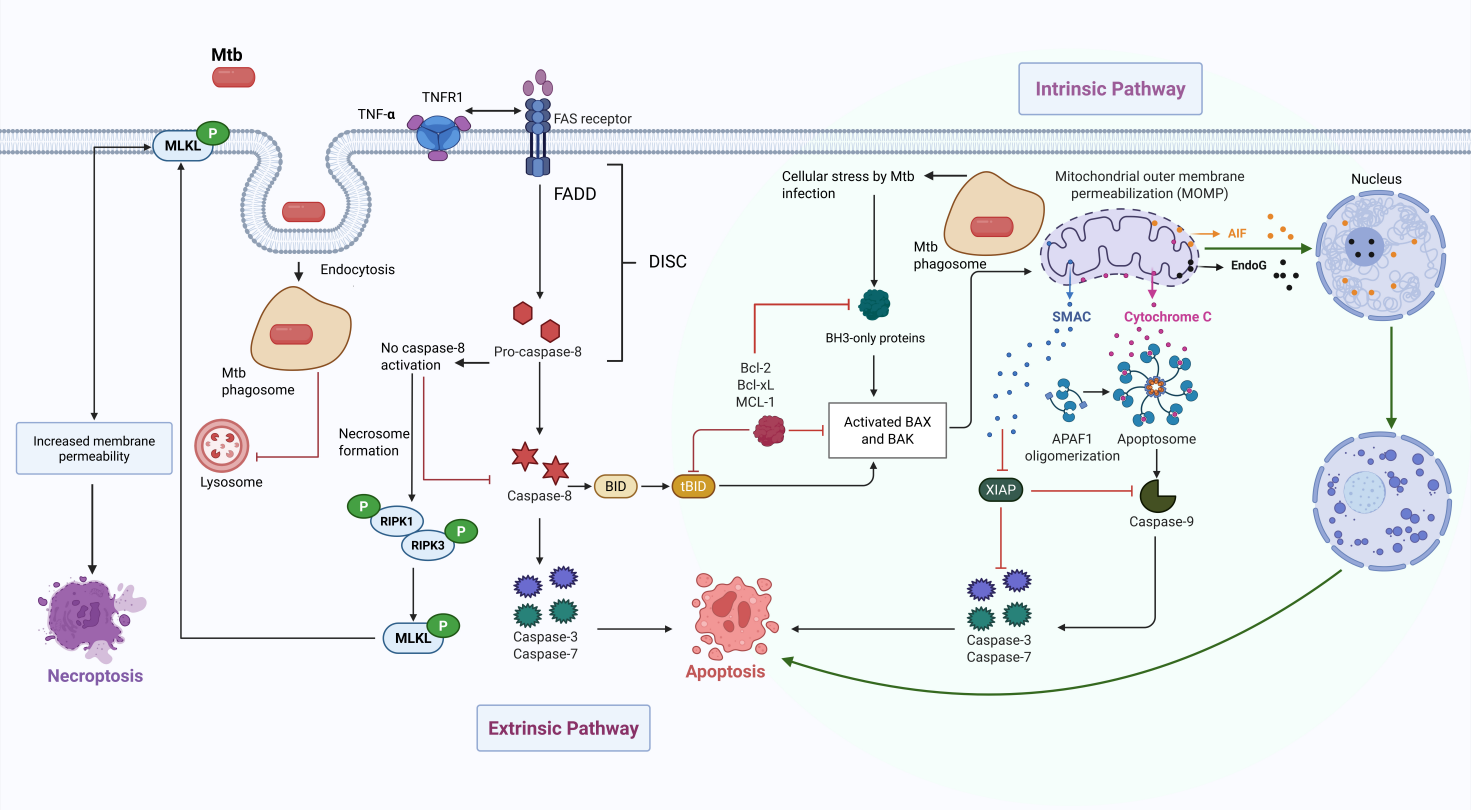
The host’s innate IR to Mtb infection triggers apoptosis and necroptosis. Apoptosis can occur via the extrinsic or intrinsic caspase-dependent and -independent pathways. Necroptosis is mediated by the phosphorylation of the MLKL protein. The black pointed head arrows indicate activating processes, while red blunt head arrows indicate inhibition. The green pointed head arrows show caspase-independent apoptotic pathways. Generated by Biorender.com.

During apoptosis, cells undergo chromatin condensation, DNA fragmentation, shrinkage, and membrane blebbing, while still maintaining membrane integrity with the formation of apoptotic bodies. Thus, this process is usually immunologically silent without activation of an inflammatory response (34). Mtb infection induces, via pattern recognition receptors, the NF-κB signaling cascade for the production and secretion of tumor necrosis factor (TNF)-α (Fig. 2). This cytokine activates macrophages to initiate extrinsic apoptosis by the transmembrane death receptor (TNF receptor 1, TNFR1) signal transduction that recruits the first apoptosis signal (Fas) receptor. This receptor interacts with the adapter Fas-associated protein with death domain (FADD), forming a death-inducing signaling complex. This leads to the recruitment of caspases to hinder bacterial growth (35, 36). The proximity of FADD allows for the autocatalytic activation of pro-caspase-8, leading to its cleavage forming their corresponding active caspases followed by the triggering of caspase-3/7 and apoptosis. For the intrinsic pathway, the mitogen-activated protein kinase (MAPK) family members, c-Jun N-terminal kinase (JNK) and p38, are involved in cellular responses to stress caused by Mtb infections that lead to the phosphorylation and ultimate activation of the BH3-only proteins, Bcl-2 associated X (BAX) and Bcl-2 antagonist killer (BAK). Moreover, the lipoprotein LpqH (Rv3763) also triggers mitochondrial outer membrane permeabilization (MOMP) and the release of apoptosis-inducing factor and cytochrome C that forms a cytoplasmic apoptosome and activates multiple caspases (37). Other pro-apoptotic factors released are endonuclease G (38) and the second mitochondrial activator of caspases. In addition, these effectors can translocate to the nucleus and initiate intrinsic apoptosis in a caspase-independent manner. Both pathways are interconnected by the action of caspase-8 that cleave the protein BH3-interacting domain (BID) into tBID. This agonist inactivates a family of proteins (Bcl-2, Bcl-xL, and Mcl-1) and initiates cell death through BAX and BAK activation resulting in MOMP.

The activation of the MAPK extracellular signal-regulated kinase pathway may have either a pro- or anti-apoptotic effect. Interleukin (IL)-10 also has this dual apoptotic role depending upon the presence of other signaling molecules. This effect is underscored by the predominant anti-inflammatory (Th2) role of IL-10 while still inducing a pro-inflammatory (Th1) response in certain cases for both mice and humans (39). IL-10 induces apoptosis, while PPE18 (Rv1196)/TLR2 pathways promote Mtb survival (40–42). This emphasizes the importance of MAPK phosphorylation in macrophages, substantiating the intracellular growth of mycobacteria. IL-10, recognized for its anti-inflammatory properties, serves as a potent suppressor of apoptotic signals, thereby enhancing host cell survival and impeding programmed cell death (43, 44). However, this pro-survival role of IL-10 needs to be carefully weighed against its potential to hinder the IR ability to eliminate intracellular pathogens (45). IL-12 promotes Th1 differentiation and indirectly impacts apoptosis by enhancing macrophage activation and cellular immunity, which leads to the apoptotic death of infected cells, restricting pathogen replication (46, 47). The pro-inflammatory cytokine IL-17 is also involved in neutrophil recruitment, driving granuloma formation and the influx of immune cells to control the mycobacterial spread (48–50). However, IL-17A has been found to protect infected macrophages by interfering with the p53 pathway and promoting Bcl-2, which increases bacterial survival inside the host (51).

The virulent strain Mtb H37Rv does induce decreased levels of apoptosis compared to the attenuated strain H37Ra (52, 53). Virulent Mtb secretes proteins that inhibit oxidative stress and/or macrophage apoptosis, such as catalase peroxidase (Rv1908c), superoxide dismutase (SodA, Rv3846), SodC (Rv0432), serine/threonine protein kinase E (PknE, Rv1743), the type I NADH dehydrogenase NuoG, and Rv3654c and Rv3655c (54–56). Moreover, the lipoprotein LpqT (Rv1016c) inhibits TLR2 signaling and apoptosis (57), while an antisense knockdown approach effectively silenced the expression of the nucleoside diphosphate kinase (*ndkA*) gene, yielding a pro-apoptotic mutant strain with an attenuated phenotype (58). Animal studies demonstrated that Mtb infection influences apoptosis in host immune cells, impacting disease progression. In mouse models, caspase-8-mediated apoptosis of infected macrophages has been shown to enhance bacterial clearance and promote adaptive IR, while inhibition of apoptosis leads to increased bacterial burden (59). Additionally, apoptosis of infected cells aids in antigen presentation to DC, enhancing cross-presentation to CD8 T cells via MHC-I and CD1 pathways (60). These *in vivo* studies demonstrated that Mtb upregulates the anti-apoptotic Bcl-2 and Rb proteins and suppresses the pro-apoptotic BAX and BAK proteins, allowing infected macrophages to survive longer with reduced caspase activity (59, 61, 62).

Thus, mycobacterial virulent strains have evolved mechanisms to suppress apoptosis, favoring necrosis to facilitate bacterial dissemination. Likewise, Mtb primarily induces necrosis rather than apoptosis in alveolar epithelial cells. However, its secreted protein ESAT-6 can trigger apoptosis in epithelial cells by inducing endoplasmic reticulum (ER) stress. The membrane-bound ER organelle affects lipid metabolism, membrane protein folding, and calcium homeostasis, and plays an important role in the activation of both the extrinsic and intrinsic pathways. ER stress emerges from the overproduction of pro-inflammatory cytokines, such as TNF-α, resulting in an increase of Ca^2+^, ROS, the unfolded protein response, and granuloma formation (63, 64). ER stress-induced apoptosis is also caused by the proteins PE_PGRS5 (Rv0297) (65) and EspC (Rv3615c), an important ESX-1 (ESAT-6 secretion system 1) secreted substrate (63). Mtb also has a counteractive mechanism to inhibit apoptosis that involves the PE_PGRS1 (Rv0109) protein to reduce the activation of protein kinase-like endoplasmic reticulum kinase (66). This highlights Mtb’s dual strategy of inhibiting or inducing apoptosis in epithelial cells depending on specific factors. Nevertheless, contradictory studies have found that the Mtb virulent strain MT103 triggers more apoptosis *in vitro* and in C57BL/6 mice as compared to Mbo Bacillus Calmette-Guerin (BCG) or the attenuated strains Mtb SO2 *phoP* (Rv0757) and the double unmarked deletion mutant MTBVAC (*phoP fadD26*), currently in phase III human clinical trials (67–71). A suggested explanation for these differences is that Mtb and *M. avium* use apoptosis of secondary-infected macrophages and epithelial cells to promote cell-to-cell spread (67, 72). However, the preponderance of the evidence is in favor of the pro-apoptotic hypothesis for live-attenuated strains based on the consistent results of multiple studies. *M. avium* also induces apoptosis through the extrinsic pathway mediated by the activation of TNF-α and Fas receptors (73, 74), while ROS activates the intrinsic mitochondrial pathway upon infection (75, 76). These pathways converge on caspase-3, executing cell death, and are further amplified by ER stress through the inositol‐requiring enzyme 1/apoptosis signal‐regulating kinase 1/JNK pathway (75). *M. avium* also produces pro-apoptotic effector proteins, including MAV_2054, which targets mitochondria to induce apoptosis (76); MAV_5183, which activates both caspase-8 and caspase-9 and promotes inflammatory cytokine secretion (77); and MAVA5_06970, which triggers rapid apoptosis in secondary-infected macrophages by interacting with osteopontin and suppressing IL-12 production (72). While apoptosis aids host defense by eliminating infected cells and limiting bacterial growth, *M. avium* can also exploit apoptotic bodies to spread and establish infection in neighboring macrophages (72, 73).

### Mtb promotes unprogrammed necrosis

Necrosis is considered host cell death without a defined genetic program of activation. Cells exhibit organelle and nuclear swelling, as well as plasma membrane swelling and rupture, leading to the release of pro-inflammatory cytoplasmic content into extracellular spaces culminating in death. Necrosis is caused by external factors, such as infectious agents, nutrition deficiency, and the environment. Later studies have shown that some necrotic processes are programmed or regulated and are defined as necroptosis, ferroptosis, and pyroptosis. Necroptosis is often mediated by death ligands and receptors (78). In one version of events (79–82), mycobacteria may activate the necroptosis program by binding TNF-α to TNFR1 (Fig. 2). This triggers the recruitment of receptor-interacting protein kinases (RIPK) 1 and 3 that are specifically serine/threonine kinases, which cross-phosphorylate each other, followed by mixed lineage kinase domain-like (MLKL) protein phosphorylation (34, 83, 84). MLKL then translocates to the membrane, inducing permeability changes that lead to necroptosis. This mechanism operates when caspase-8 is inhibited culminating in necrosome formation, comprising RIPK1, RIPK3, and other factors (85–87). A subsequent study showed that the tuberculosis necrotizing toxin acts as an NAD^+^ hydrolase activating RIPK3 and MLKL, but not RIPK1, with the final outcome of pore formation and cell lysis (88). In addition, RIPK1 and NF-κB signaling in dying cells promotes cross-priming of CD8(+) T cells (89), and Bcl-xL mediates RIPK3-dependent necroptosis in Mtb-infected macrophages (84). Complicating the analysis, necroptosis may be activated as well with the involvement of RIPK1, RIPK3, MLKL, and the cytokine IL-4 (90, 91). IL-4 induces the Th2 response, and it is typically associated with anti-inflammatory and tissue repair functions.

Later studies revealed that necroptosis is not the primary cell lysis pathway. The main mycobacterial effectors are transported by the type VII secretion system and other microbial components. Nonetheless, experiments with human and murine BMDM, and *in vivo* experiments with C57BL/6 mice, clearly demonstrated that the necroptosis activation is not fundamental to the necrotic process induced by Mtb (92). Thus, virulent Mtb induces unprogrammed necrosis from the host point of view (13). Mtb exploits macrophage cell death to foster an environment conducive to proliferation within the host. By inducing necrosis, a virulent Mtb strain evades macrophage defenses, thwarting anti-mycobacterial protective mechanisms (84, 93, 94). Infected macrophages undergoing necrosis can release intracellular Mtb into extracellular spaces due to plasma membrane breakdown. This process involves lysis caused by CpnT (Rv3903c), EsxA, and PDIM to promote extracellular replication and suppression of the host IR (95). As previously mentioned, a virulent Mtb strain actively suppresses apoptosis in macrophages via NuoG and SecA2 (Rv1821), opting for the induction of a necrotic process (55, 93, 96–98).

### Mtb activates ferroptosis via oxidative stress and lipoxygenation of arachidonic acid

**Fig 3:**
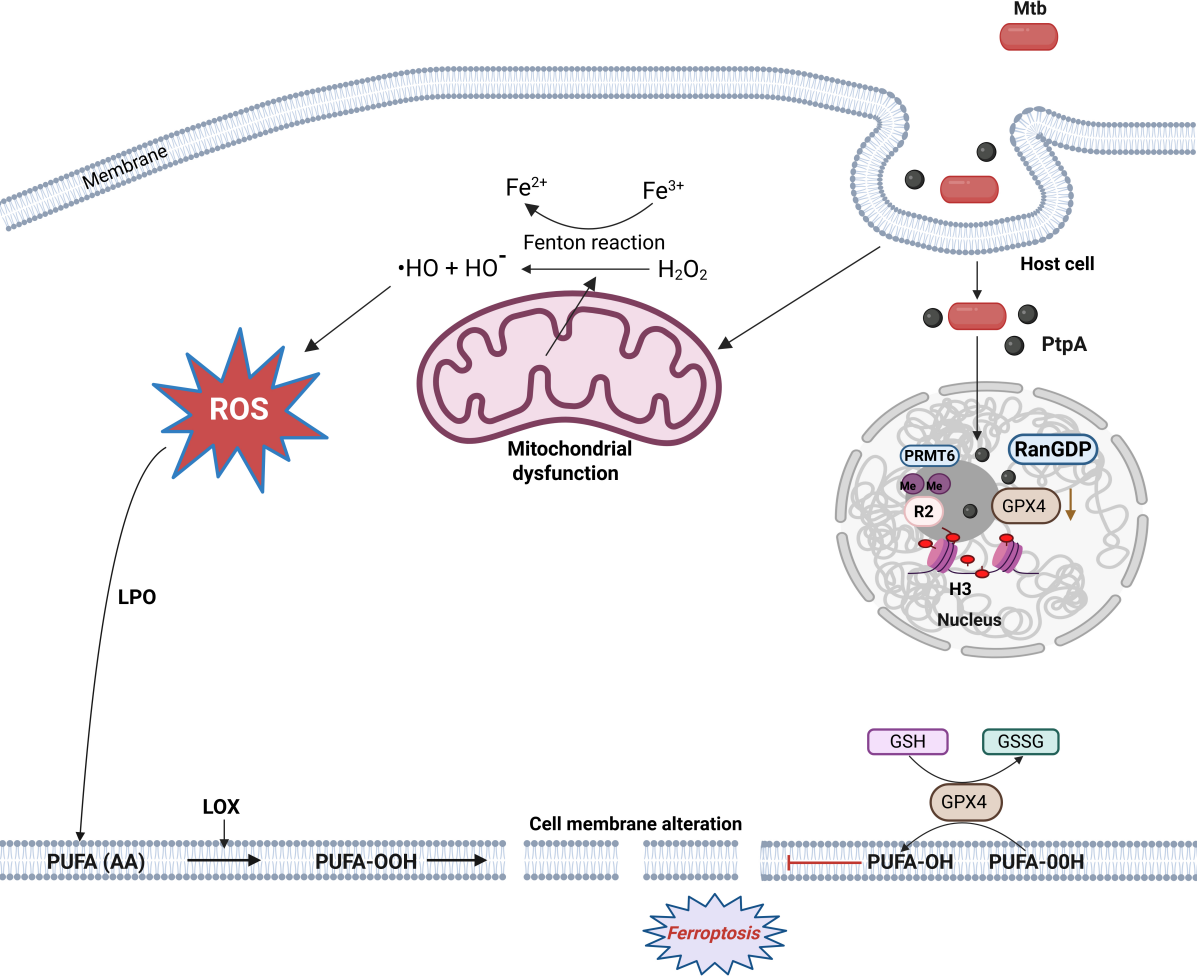
Events leading to ferroptosis by oxidative damage of cell membrane fatty acids. The black pointed head arrows indicate activating processes, while red blunt head arrows indicate inhibition. Generated by Biorender.com.

Ferroptosis, involved in the pathogenesis of several infectious and noninfectious diseases, is a type of regulated cell death with necrotic morphology characterized by iron-dependent lipid peroxidation (LPO) caused by oxidative stress (99, 100). During Mtb infection, the PtpA effector enters the host cell nucleus and interacts with the GDP-bound Ras-related nuclear protein (Ran) GTPase (Ran-GDP) (Fig. 3). This promotes the dimethylation of histone H3 arginine 2 (H3R2me2a) via the protein arginine methyltransferase 6, leading to the reduction of glutathione peroxidase 4 (GPX4) transcription and the initial stimulation of ferroptosis (101). GPX4 is a phospholipid hydroperoxidase that protects cells against membrane LPO. In addition, the peptidyl-prolyl isomerase A (Rv0009) interacts with the integrin signaling and ferroptotic pathways promoting microbial persistence and dissemination (102). Moreover, Mtb induces the Fenton reaction in mitochondria, yielding highly reactive hydroxyl radicals (˙HO) and other ROS products from hydrogen peroxide (H_2_O_2_) and ferric iron (Fe^3+^). This reaction leads to mitochondrial dysfunction, LPO, and depletion of glutathione (GSH) and GPX4 with a concomitant increase in glutathione disulfide (GSSG).

Arachidonic acid (AA) is the main polyunsaturated fatty acid (PUFA) involved in cell membrane integrity that undergoes LPO. Moreover, AA metabolism via cyclooxygenases or 5-lipoxygenases leads to the formation of the eicosanoids prostaglandin E_2_ that protects the mitochondria and induces cell membrane repair, and lipoxin A_4_ that is involved in inflammatory responses and ferroptosis, respectively (53). Evidently, eicosanoids emerge as pivotal regulators balancing between apoptosis and ferroptosis by modulating both innate and adaptive responses during Mtb infection (103). Notably, an excess of the pro-inflammatory eicosanoid leukotriene B4 leads to heightened TNF-α production (104). This phenomenon subsequently increases host susceptibility (105). Another study identified the secretory protein Rv1324 as having antioxidant activity, increasing *Mycobacterium smegmatis* (Msm, a low pathogenicity model system) resistance to RNS and ROS. This protein activates the ferroptosis pathway during infection, promoting mycobacterial survival and causing tissue damage (106).

### Mtb prevents pyroptosis in macrophages while attenuated strains do not

**Fig 4:**
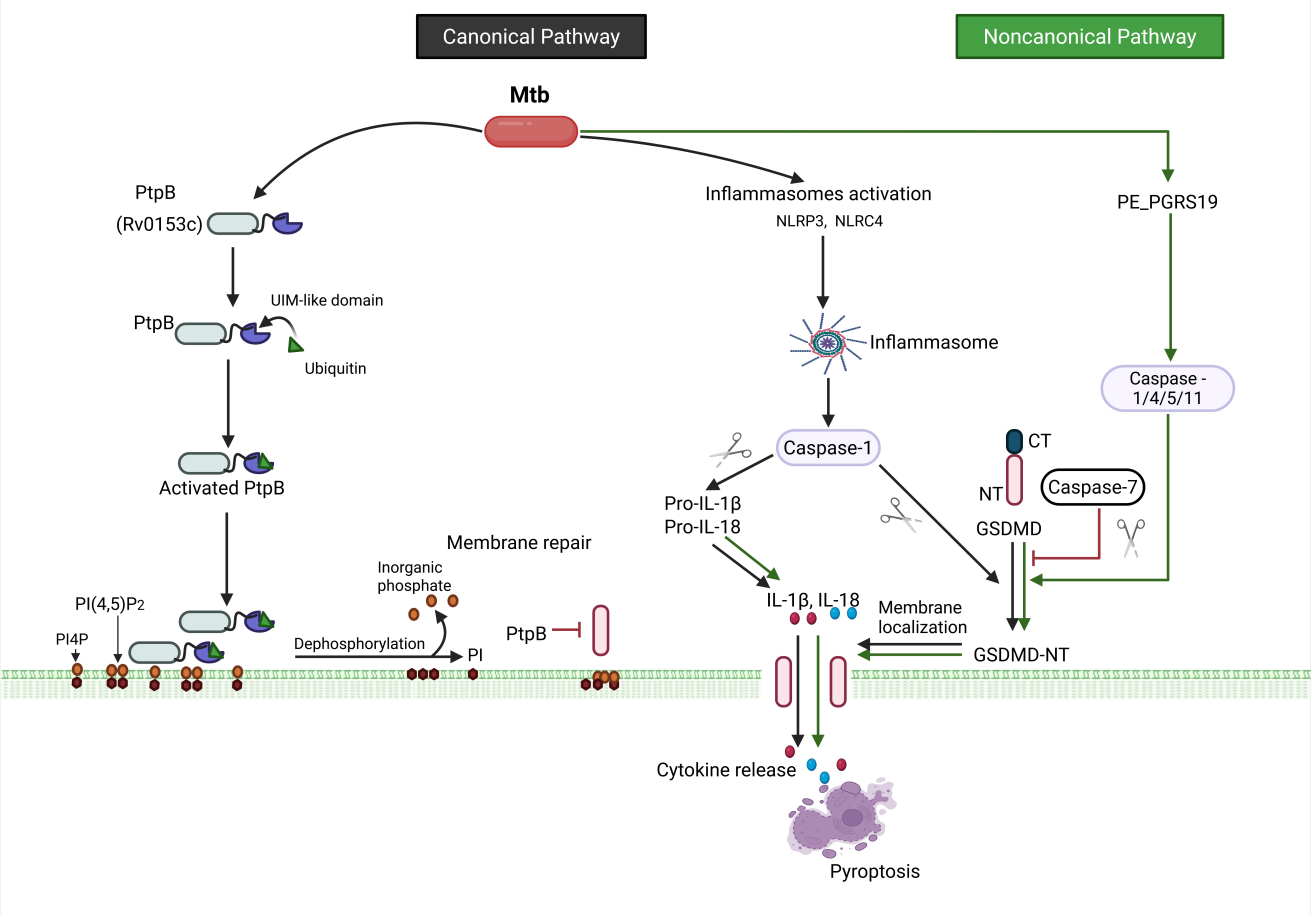
Mtb infection leads to the activation of NLRP3 and NLRC4 inflammasomes causing pyroptosis, while the Mtb PtpB protein inhibits this process. The black (canonical pathway) or green (noncanonical pathway) pointed head arrows indicate activating processes, while red blunt head arrows indicate inhibition. Generated by Biorender.com.

Pyroptosis is a distinct programmed cell death mechanism that can be differentiated from the other cell death processes. Pyroptosis involves the inflammatory caspases (−1, −4, −5, and −11) and six gasdermin family proteins (GSDM A-E and DFNB59). A main difference between necroptosis and unprogrammed necrosis is that cell swelling and lysis are mediated by GSDM, as opposed to mycobacterial effectors, which determine pore formation in the cytoplasmic membrane. In addition, lower levels of pyroptosis may play a crucial role in innate immunity against infections (107). Pyroptosis can be triggered through the canonical pathway, which involves inflammasomes: NLRP3 (NOD-like receptor family pyrin domain containing 3) and NLRC4 (NOD-like receptor family CARD domain containing 4) that activate caspase-1, while the noncanonical pathway involves caspase-1/4/5/11 (Fig. 4). Both pathways lead to the cleavage of gasdermin D (GSDMD) by caspases, releasing the N-terminal fragment (GSDMD-NT) that forms pores in the cell membrane resulting in lysis and the release of pro-inflammatory cytokines like IL-1β and IL-18 (108).

A study demonstrated that NLRP3 activates pyroptosis causing severe damage, including organelle destruction with content leakage and large plasma membrane disruptions accompanied by mitochondrial depolarization (109). This study suggested that pyroptosis allows for Mtb spread; however, most experiments were conducted with cell lines. Indeed, later studies suggested a different mechanism implicating virulent Mtb in the evasion of pyroptosis and the subsequent host IR. Mtb secretes the effector protein phosphotyrosine phosphatase (PtbB, Rv0153c), which interacts with the host ubiquitin and disrupts GSDMD-NT membrane localization by dephosphorylating phosphatidylinositol-4-monophosphate (PI4P) and phosphatidylinositol-(4, 5)-bisphosphate (PI(4, 5)P_2_) in the cell membrane. In addition, the protein zinc metalloprotease (Zmp1, Rv0198c) prevents inflammasome activation and IL-1β processing. Thus, cytokine release and macrophage pyroptosis are inhibited (110, 111). This study was substantiated by experiments with murine BMDM and C57BL/6 mice. Mtb metabolism also plays a key role since its dysregulation increases NLRP3 inflammasome activation in macrophages, leading to pyroptosis and the induction of the adaptive IR, including increased T cell activation and proliferation (112). In this study, a recombinant Mbo BCG Danish 1331 strain that behaves similarly in cell death processes to virulent Mtb was developed. Transposon mutants were isolated with increased pyroptotic properties that mapped in metabolic and conserved hypothetical proteins that inhibit cell death. These were called functioning death repressor (*fdr*) genes (*arcA*, Rv1001; Rv1204c; Rv1831; Rv2795c; Rv3727; and *tcrX*, Rv3765c). The attenuated Mtb strain H37Ra also increased pyroptosis under similar conditions. These results suggest that avirulent strains promote pyroptosis to prevent Mtb dissemination. These results will place pyroptosis more akin to apoptosis than necrosis. Moreover, the GSDM pores can be repaired by caspase-7, leading to the production of ceramide, allowing host cells to survive (113).

## VIRULENCE FACTORS INVOLVED IN CELL DEATH PATHWAYS FOR OTHER MYCOBACTERIAL SPECIES AND THEIR ATTENUATED MUTANTS

Cell death mechanisms are important in other mycobacterial human and animal pathogens, though the specific modalities depend upon the relevant host/pathogen interactions. For comparative purposes of the virulence factors described in this review, a National Center for Biotechnology Information (NCBI) Basic Local Alignment Search Tool Protein (BLASTp) search was conducted with Mtb H37Rv (GenBank AL123456.3) to identify the gene homologs for Mbo AF2122-97 (GenBank LT708304.1), Mbo BCG str. Pasteur 1173P2 (GenBank AM408590.1), *M. avium* 104 (GenBank CP000479.1), Map K-10 (GenBank AE016958.1), and Msm mc^2^155 (GenBank CP000480.1) (Table S1). The *M. avium* 104 current NCBI taxonomic nomenclature was previously referred to as *M. avium* subsp. *hominissuis* 104 (4). The “subsp. *hominissuis*” was proposed to differentiate bacteria discovered in humans and pigs from bird isolates, but *M. avium* 104 and other clinical isolates have distinct genotypic characteristics. In addition, Table S2 displays an NCBI protein Conserved Domain Database (CDD) search for each Table S1 gene homolog. This search becomes important since similar domains indicate the same functionality. Even though protein domain sequences can be identical, variations in adjacent amino acid sequences can lead to different architectural structures and functionalities based on protein folding (114).

The genomes of Mtb, virulent Mbo, and Mbo BCG are highly similar (>99.9%). Mtb is a human-adapted pathogen, Mbo is a zoonotic pathogen, and Mbo BCG is the only approved attenuated TB vaccine. All Mtb virulence factors listed in Table S1 have a corresponding gene homolog for virulent and attenuated Mbo. All proteins have >93.1% identity except the following: Rv0222 (35.1% with BQ2027_MB1099C and 30.9% with BCG_3445), Rv1468c (69.1% with BQ2027_MB3626C and BCG_3660c), Rv2741 (66.9% with BQ2027_MB3626C), and Rv3875 (34.6% with BCG_3511c). This is consistent with the well-known fact that BCG has a deletion in the RD1 region, and the homology is due to another protein (EsxU) annotated as hypothetical. The CDD search (Table S2) also discovered that all mycobacterial strain homologs for Rv1181, Rv1182, Rv2181, Rv3310, Rv3655c, Rv3820c, and Rv3824c had “No Specific Hits.” Even though Rv1180 had a conserved domain, the homologs in Mbo, Mbo BCG, MAV_1321, and MSMEG_4727 had “No Specific Hits.” “No Conserved Domains” were determined for Rv1831 and Rv3654c and their Mbo and Mbo BCG homologs, while Rv1759c did have a conserved domain, and both Mbo strains did not.

*M. avium* subspecies have genome similarities ranging from 72% to 95% with Mtb. A BLASTp with Rv1831 and Rv3615c and *M. avium* 104 found “No Gene Identified,” while the same was true for Rv0198c, Rv3615c, and Rv3654c and Map. Also, the percent identity varies from 25.8% for an oxidoreductase (MAV_4796 and MAP_3849 vs Rv3727) to about 94% for two transcriptional regulatory proteins (MAV_0701 and Rv0757; MAP_0259 and Rv_3765c). In addition, the CDD search had “No Specific Hits” for the *M. avium* and Map homologs of Rv1184c, Rv1185c, Rv3451, and Rv3727; *M. avium* homolog of Rv1204c and Rv2930; and the Map homolog of Rv1183 and Rv3823c. “No Conserved Domains” were found for Rv3654c and Rv1831 and their homologs MAV_0514 and MAP_1544, respectively. Mtb and Msm have a genome similarity of only about 75% since the latter lacks many of the virulence factors present in Mtb. There were the most Mtb proteins found as “No Gene Identified” for Msm: Rv0109, Rv0297, Rv1068c, Rv1468c, Rv1635c, Rv1759c, Rv1831, Rv2741, Rv2878c, Rv3310, Rv3615c, and Rv3727. The lowest percent identity was 24.5% for MSMEG_1870 and Rv3903c, with 78% of the proteins having <75% identity. “No Specific Hits” were determined for the Msm homologs of Rv1180, Rv1181, Rv1184c, Rv2181, Rv3451, Rv3655c, Rv3820c, and Rv3824c.

In a study on Mbo BCG using peripheral blood samples from pulmonary and extra-pulmonary TB patients, transcriptomic analysis revealed the downregulation of GPX4 but an increase in heme oxygenase-1 protein (HO-1) gene expression (115). These findings were replicated in C57BL/6 mice infected with Mbo BCG. At 30 days post-infection, both lung tissues revealed similar up- and downregulation of these proteins. Results in RAW264.7 cells revealed that the siRNA knockdown of HO-1 led to an increase of intracellular ferrous iron, iron autophagy factors, and ROS along with decreased levels of the ferroptosis suppressor protein 1 and GPX4, leading to a release of intracellular bacteria. In contrast, the upregulation of HO-1 suppresses ferroptosis and bacterial release in the infected macrophages by fine-tuning iron and ROS levels. Another study found that infection with virulent Mbo causes ER stress, which in turn activates an interferon gene stimulator that recruits a kinase to activate the interferon regulatory factor 3 (IRF3) and eventually cell death (116). This leads to the triggering of caspases, mitochondrial damage, and eventual apoptosis. Blocking ER stress or IRF3 signaling significantly reduced both apoptosis and the host’s ability to control intracellular bacteria.

The inverse correlation between virulence and apoptosis was reported in *M. avium* (76). MAV_2054 induces the production of ROS, disrupts mitochondrial membrane potential, and triggers cytochrome release activating the intrinsic apoptotic pathway. Interestingly, recombinant Msm expressing MAV_2054 had reduced survival in macrophages and C57BL/6 mice. However, the heterologous expression and its regulation in Msm casts doubts on these findings. It is possible that this protein plays a role in nonphagocytic cells favoring invasion of epithelial cells, as shown in Map for the 35-kDa major membrane protein MAP_2121c that is 100% identical to MAV_2054 (117). Thus, the dichotomy of favoring apoptosis in macrophages and epithelial cells reveals that apoptosis is a host defense mechanism when macrophages are involved, as it restricts intracellular replication. In contrast, apoptosis in epithelial cells would favor pathogen spread.

The infection of RAW264.7 macrophages with Mbo BCG leads to the downregulation of fatty acid-binding protein 4 and LincRNA-Cox2, β-oxidation of fatty acids, and ROS generation (118, 119). Msm binds the TLR2 that triggers a cytosolic Ca^2+^ surge and the release of superoxide, nitric oxide, and TNF-α (120). In our recent study with Map, we observed that RAW246.7 macrophage cells infected with the wild-type strain do not undergo higher apoptotic levels compared to the uninfected controls (121). However, another study that separated bystander from infected primary macrophages observed inhibition of apoptosis (122). Nonetheless, in both studies, attenuated mutant strains were clearly pro-apoptotic. We also observed that attenuated strains undergo significant levels of apoptosis followed by a necrotic process upon further incubation, probably a terminal step in cell culture without major physiological implications (121). This may explain some necrotic events previously observed (69). Thus, the inverse relationship between apoptosis and strain virulence can be related to differences in methodologies and cell types: primary vs immortal cell lines, resident alveolar vs blood-derived macrophages, and the proportions of anti-apoptotic M1 and pro-apoptotic M2 macrophages (123).

## THERAPEUTIC IMPLICATIONS

Proposed host-directed therapies promote cell death pathways that are favorable, including autophagy, apoptosis, and pyroptosis. In contrast, these therapies may seek the inhibition of necrosis and ferroptosis that contribute to disease progression. Autophagy modulators can be potential targets in TB treatment to regulate the IR and prevent immune suppression during active high-dose Mtb infections (32). Proposed drug therapies to promote autophagy include the AMP-activated protein kinase pathway, the inhibition of mTOR using rapamycin (directly) and metformin (indirectly), and vitamin D receptor signaling (124). Regarding apoptosis activation, the following therapies are being examined: vitamin D supplementation, TNF-α modulation, and the induction of IFN-γ (125–127). Other strategies involve anti-apoptotic inhibitors that include Bcl-2 (e.g., venetoclax), mTOR (e.g., rapamycin), immunotherapy checkpoint (e.g., PD-1/PD-L1 blockers), and epigenetic modulators (e.g., histone deacetylase and vorinostat) (128–130). Recently, the pro-apoptotic drug navitoclax was shown to be an effective host-directed TB therapy since navitoclax induces apoptosis in key immune cells, such as CD68^+^ and CD11b^+^, within lesions and reduces fibrosis in mice (131).

Therapies that promote pyroptosis are inflammasome (e.g., NLRP3 and AIM2), GSDMD, and caspase activators, TLR2/4/9 agonists, natural prebiotic compounds (e.g., berberine and curcumin), probiotic bacterial strains (e.g., *Lactobacillus* and *Bifidobacterium*), and the previously mentioned checkpoint blockers, genetic engineering, and nanoparticles (132–135). In contrast, necrosis inhibition has also been suggested, such as the use of corticosteroids, metformin, vitamin D, anti-TNF compounds, and phosphodiesterase inhibitors. Ferroptosis inhibition has been sought by repurposing anti-TB drugs such as isoniazid and rifampicin, as well as gene therapy approaches to overexpress GPX4 and RNAi to inhibit acyl-CoA synthetase, and by targeting mitochondrial dysfunction (136–138). In addition, the natural compound galangin inhibits PI3K and related signaling pathways (139). Furthermore, the ferroptosis inhibitor ferrostatin-1 also decreases Mtb bacterial loads and pulmonary necrosis in infected C57BL/6J mice (140). The potential therapeutic implications of exploiting attenuated mycobacterial vaccines to induce autophagy and apoptosis contribute to harnessing the unique immune-stimulatory properties of attenuated strains. Thus, targeted interventions that drive host immune cells toward apoptosis induction can be implemented. This strategy not only aids in reducing the bacterial burden but also helps in preventing disease progression and the establishment of chronic infections.

## CONCLUDING REMARKS

In this review, we summarize the main findings regarding the role of cell death pathways in mycobacterial infections. The preponderance of experimental results clearly indicates that mycobacterial pathogens inhibit apoptosis and pyroptosis, while promoting necrosis and ferroptosis. However, the extent to which these processes are affected depends on what virulence factors are present in the different species (Tables S1 and S2). The role of autophagy is more subtle as its machinery is needed to control Mtb infections. In contrast, attenuated strains promote apoptosis and pyroptosis as a way to induce protective immunity. These conclusions are based on a careful analysis of the literature highlighting studies using mycobacterial deletion mutants, mutated mice/cell lines, and primary cells. We discussed some contradictions in the field and attempted to provide a resolution based on the majority of the evidence.

Despite the exciting prospects, several challenges remain. The mechanisms by which attenuated strains specifically trigger apoptosis need further elucidation. Additionally, the potential for unintended IRs or side effects requires careful evaluation. The selection of optimal attenuated strains, tailored to the specific mycobacterial infection, is a critical consideration moving forward. Translating these findings into clinical applications demands rigorous investigations to ensure safety and efficacy. The battle between apoptosis and necrosis during mycobacterial infections is a decisive factor in shaping disease outcomes. Attenuated mycobacterial strains, with their propensity to drive apoptosis, offer a novel approach for advancing vaccine development and immunomodulatory therapies. Thus, pro-apoptotic strains are usually good vaccine candidates. An example is the VPM1002 vaccine that has reached phase III clinical trials (141). In this strain, the listeriolysin O was added to promote phagosomal lysis and enhance polyfunctional CD4^+^ and CD8^+^ T cells, including IL-17^+^ production (142), and its immunogenicity was further improved by the deletion of *nuoG*, yielding the pro-apoptotic strain vaccine candidate (143). Insights from these investigations promise to address mycobacterial infections and contribute to the broader arsenal against infectious diseases.

## SUPPLEMENTAL MATERIAL

Table S1 iai.00401-25-s0001.docx BLASTp host factors.

Table S2 iai.00401-25-s0002.docx CDD host factors.

ASM does not own the copyrights to Supplemental Material that may be linked to, or accessed through, an article. The authors have granted ASM a non-exclusive, world-wide license to publish the Supplemental Material files. Please contact the corresponding author directly for reuse.
