# Supplementary material for: Cell death pathways in response to Mycobacterium tuberculosis and other mycobacterial infections
Source: Infect Immun. 2025 Sep 9;93(10):e00401-25. doi: 10.1128/iai.00401-25 (PMC12519786; doi:10.1128/iai.00401-25)
Supplement: Table S1 — BLASTp host factors. [file iai.00401-25-s0001.docx]

**Supplemental Table S1.** Mycobacterial virulence factors involved in cell death pathways. For each *Mycobacterium* listed with taxonomy identification and GenBank numbers, a National Center for Biotechnology Information (NCBI) Basic Local Alignment Search Tool^a^ Protein (BLASTp) search^b^ identified the gene homologs^c^. In addition, an accession number, Expect value^d^, Identity, and bits Score^e^ are provided.

| ***M. tuberculosis* H37Rv**  **taxid:83332**  **GenBank**  **AL123456.3** | ***M. bovis* AF2122-97**  **taxid:233413**  **GenBank**  **LT708304.1** | ***M. bovis* BCG str. Pasteur 1173P2**  **taxid:410289**  **GenBank**  **AM408590.1** | ***M. avium* 104**  **taxid:243243**  **GenBank**  **CP000479.1** | ***M. avium* subsp. *paratuberculosis* K-10**  **taxid:262316**  **GenBank**  **AE016958.1** | ***M. smegmatis* mc^2^155**  **taxid:246196**  **GenBank**  **CP000480.1** |
| --- | --- | --- | --- | --- | --- |
| Rv0009  PpiA  Probable iron-regulated peptidyl-prolyl cis-trans isomerase A | BQ2027_MB0009  WP_003400321.1  P65763.1  2e-135 Expect value  100% Identity  373 bits Score | BCG_0009  WP_003400321.1  CAL69993.1  2e-135 Expect value  100% Identity  373 bits Score | MAV_0013  ABK66705.1  1e-109 Expect value  90.3% Identity  308 bits Score | MAP_0011  WP_003876785.1  3e-113 Expect value  89.6% Identity  317 bits Score | MSMEG_0024  WP_003891350.1  ABK70605.1  2e-105 Expect value  89.6% Identity  298 bits Score |
| Rv0109  PE_PGRS1  PE-PGRS family protein | BQ2027_MB0113  WP_003909149.1  0.0 Expect value  99.8% Identity  826 bits Score | BCG_0142  WP_003909149.1  0.0 Expect value  99.8% Identity  826 bits Score | MAV_1346  WP_010949655.1  ABK65871.1  6e-17 Expect value  51.1% Identity  78.6 bits Score | MAP_4144  AAS06694.1  2e-29 Expect value  61.0% Identity  112 bits Score | No Gene Identified |
| Rv0153c  PtbB (a.k.a MPtpB)  Phosphotyrosine protein phosphatase | BQ2027_MB0158C  WP_010950352.1  0.0 Expect value  99.6% Identity  547 bits Score | BCG_0189c  WP_010950352.1  CAL70173.1  0.0 Expect value  99.6% Identity  547 bits Score | MAV_5145  WP_009979776.1  ABK65139.1  5e-151 Expect value  76.8% Identity  421 bits Score | MAP_3568c  AAS06118.1  2e-82 Expect value  78.1% Identity  242 bits Score | MSMEG_0100  WP_011726668.1  ABK73175.1  4e-99 Expect value  56.3% Identity  289 bits Score |
| Rv0198c  Zmp1  Probable zinc metalloprotease | BQ2027_MB0204C  WP_003401172.1  0.0 Expect value  100% Identity  1350 bits Score | BCG_0235c  WP_003401172.1  0.0 Expect value  100% Identity  1350 bits Score | MAV_4977  WP_011726364.1  0.0 Expect value  80.5% Identity  1068 bits Score | No Gene Identified | MSMEG_0234  WP_011726765.1  ABK69870.1  0.0 Expect value  77.0% Identity  1003 bits Score |
| Rv0222  echA1  enoyl-CoA hydratase | BQ2027_MB1099C  [WP_003405712.1](https://www.ncbi.nlm.nih.gov/protein/WP_003405712.1?report=genbank&log$=protalign&blast_rank=3&RID=6XG7NYCR014)  [P64017.1](https://www.ncbi.nlm.nih.gov/protein/P64017.1?report=genbank&log$=protalign&blast_rank=3&RID=6XG7NYCR014)  6e-36 Expect value  35.1% Identity  125 bits Score | BCG_3445  [CAL73434.1](https://www.ncbi.nlm.nih.gov/protein/CAL73434.1?report=genbank&log$=prottop&blast_rank=8&RID=6XGEYTD7014)  1e-20 Expect value  30.9% Identity  86.3 bits Score | MAV_4951  [ABK65185.1](https://www.ncbi.nlm.nih.gov/protein/ABK65185.1?report=genbank&log$=prottop&blast_rank=1&RID=6XGPS6WU016)  1e-130 Expect value  84.9% Identity  368 bits Score | MAP_3658  [AAS06208.1](https://www.ncbi.nlm.nih.gov/protein/AAS06208.1?report=genbank&log$=prottop&blast_rank=2&RID=6XGUYP70016)  4e-130 Expect value  84.9% Identity  366 bits Score | MSMEG_2227  [WP_011728235.1](https://www.ncbi.nlm.nih.gov/protein/WP_011728235.1?report=genbank&log$=protalign&blast_rank=1&RID=6XGYRADD016)  [ABK70079.1](https://www.ncbi.nlm.nih.gov/protein/ABK70079.1?report=genbank&log$=protalign&blast_rank=1&RID=6XGYRADD016)  2e-114 Expect value  65.6% Identity  327 bits Score |
| Rv0256c  PPE2  PPE family protein | BQ2027_MB0262C  WP_003401347.1  0.0 Expect value  100% Identity  1083 bits Score | BCG_0294c  WP_003401347.1  0.0 Expect value  100% Identity  1083 bits Score | MAV_1329  ABK65643.1  1e-158 Expect value  58.9% Identity  462 bits Score | MAP_2595  AAS04912.1  6e-130 Expect value  65.5% Identity  389 bits Score | MSMEG_0619  WP_011727070.1  ABK70912.1  9e-55 Expect value  72.1% Identity  191 bits Score |
| Rv0288  EsxH  Low molecular weight protein antigen 7 | BQ2027_MB0296  WP_003401514.1  P0A569.2  2e-66 Expect value  100% Identity  192 bits Score | BCG_0328  WP_003401514.1  2e-66 Expect value  100% Identity  192 bits Score | MAV_4865  ABK67570.1  1e-53 Expect value  79.2% Identity  159 bits Score | MAP_3784  WP_003879185.1  AAS06334.1  2e-53 Expect value  79.2% Identity  159 bits Score | MSMEG_0621  WP_003892041.1  ABK72724.1  3e-48 Expect value  75.8% Identity  146 bits Score |
| Rv0297  PE_PGRS5  PE-PGRS family protein | BQ2027_MB0305  WP_003401546.1  0.0 Expect value  97.5% Identity  967 bits Score | BCG_0337  WP_003401546.1  0.0 Expect value  97.5% Identity  967 bits Score | MAV_1346  WP_010949655.1  ABK65871.1  2e-14 Expect value  48.9% Identity  71.6 bits Score | MAP_4144  AAS06694.1  3e-24 Expect value  56.7% Identity  98.2 bits Score | No Gene Identified |
| Rv0410c  PknG  Serine/threonine-protein kinase | BQ2027_MB0418C  WP_003402100.1  P65729.1  0.0 Expect value  100% Identity  1523 bits Score | BCG_0449c  WP_003402100.1  0.0 Expect value  100% Identity  1523 bits Score | MAV_4751  WP_011726224.1  ABK65526.1  0.0 Expect value  85.3% Identity  1265 bits Score | MAP_3893c  WP_010950156.1  AAS06443.1  0.0 Expect value  85.6% Identity  1269 bits Score | MSMEG_0786  WP_011727190.1  ABK71095.1  0.0 Expect value  79.6% Identity  1197 bits Score |
| Rv0432  SodC  Periplasmic superoxide dismutase | BQ2027_MB0440  WP_003402198.1  P0A609.1  8e-175 Expect value  100% Identity  477 bits Score | BCG_0471  WP_003402198.1  CAL70456.1  8e-175 Expect value  100% Identity  477 bits Score | MAV_4722  WP_009979378.1  ABK68594.1  2e-114 Expect value  72.9% Identity  324 bits Score | MAP_3921  WP_003873788.1  AAS06471.1  2e-114 Expect value  72.9% Identity  324 bits Score | MSMEG_0835  WP_011727227.1  ABK75751.1  1e-101 Expect value  68.9% Identity  293 bits Score |
| Rv0757  PhoP  Possible two component system response transcriptional positive regulator | BQ2027_MB0780  WP_003403867.1  0.0 Expect value  100% Identity  496 bits Score | BCG_0809  WP_003403867.1  CAL70795.1  0.0 Expect value  100% Identity  496 bits Score | MAV_0701  WP_003875752.1  2e-164 Expect value  94.2% Identity  452 bits Score | MAP_0591  WP_010948903.1  AAS02908.1  8e-164 Expect value  93.7% Identity  450 bits Score | MSMEG_5872  WP_011730856.1  ABK73640.1  1e-157 Expect value  92.6% Identity  435 bits Score |
| Rv1001  ArcA  Probable arginine deiminase | BQ2027_MB1028  WP_003405169.1  P63552.1  0.0 Expect value  100% Identity  804 bits Score | BCG_1058  WP_003405169.1  A1KHD9.1  0.0 Expect value  100% Identity  804 bits Score | MAV_1125  WP_009975265.1  A0QBT9.1  0.0 Expect value  89.7% Identity  720 bits Score | MAP_0942  AAS03259.1  0.0 Expect value  89.5%Identity  720 bits Score | MSMEG_5448  ABK73603.1  0.0 Expect value  80.7% Identity  643 bits Score |
| Rv1016c  LpqT  Probable conserved lipoprotein | Q2027_MB1044C  Q7U0V0.1  2e-139 Expect value  100% Identity  388 bits Score | BCG_1073c  CAL71060.1  2e-139 Expect value  100% Identity  388 bits Score | MAV_0054  WP_011723312.1  ABK64724.1  2e-20 Expect value  34.3% Identity  85.9 bits Score | MAP_0047c  AAS02364.1  2e-20 Expect value  34.3% Identity  86.3 bits Score | MSMEG_5429  WP_011730528.1  ABK71494.1  7e-82 Expect value  56.2% Identity  241 bits Score |
| Rv1068c  PE_PGRS20  PE-PGRS family protein | BQ2027_MB1097C  SIT99696.1  3e-70 Expect value  99.5% Identity  234 bits Score | BCG_1126c  WP_011799151.1  CAL71113.1  2e-70 Expect value  99.5% Identity  234 bits Score | MAV_2923  ABK66202.1  1e-08 Expect value  51.0% Identity  49.3 bits Score | MAP_4144  AAS06694.1  3e-17 Expect value  47.9% Identity  77.4 bits Score | No Gene Identified |
| Rv1159  PimE  Mannosyltransferase;  PIM (phosphatidyl-inositol mannoside) | BQ2027_MB1190  WP_003900282.1  0.0 Expect value  100% Identity  839 bits Score | BCG_1220  WP_003900282.1  0.0 Expect value  100% Identity  839 bits Score | MAV_1298  ABK65752.1  0.0 Expect value  77.8% Identity  592 bits Score | MAP_2624c  WP_003878499.1  0.0 Expect value  75.5% Identity  601 bits Score | MSMEG_5149  AFP41462.1  A0R2K8.2  0.0 Expect value  70.8% Identity  538 bits Score |
| Rv1180  Pks3  Probable polyketide beta-ketoacyl synthase;  DAT (diacyltrehalose) | BQ2027_MB1213  WP_010950502.1  0.0 Expect value  100% Identity  989 bits Score | BCG_1243  WP_011799162.1  0.0 Expect value  100% Identity  988 bits Score | MAV_1321  ABK65112.1  0.0 Expect value  80.3% Identity  731 bits Score | MAP_2604c  AAS04921.1  0.0 Expect value  82.6% Identity  814 bits Score | MSMEG_4727  WP_011730042.1  A0R1E8.1  0.0 Expect value  71.0% Identity  703 bits Score |
| Rv1181  Pks4  Probable polyketide beta-ketoacyl synthase;  DAT (diacyltrehalose) | BQ2027_MB1213  WP_010950502.1  0.0 Expect value  99.9% Identity  3165 bits Score | BCG_1243  WP_011799162.1  0.0 Expect value  99.8% Identity  3163 bits Score | MAV_1321  ABK65112.1  0.0 Expect value  74.7% Identity  2290 bits Score | MAP_3764c  AAS06314.1  0.0 Expect value  65.5% Identity  1983 bits Score | MSMEG_4727  WP_011730042.1  A0R1E8.1  0.0 Expect value  62.4% Identity  1878 bits Score |
| Rv1182  PapA3  Probable conserved polyketide synthase associated protein; DAT (diacyltrehalose) | BQ2027_MB1214  WP_003898759.1  0.0 Expect value  100% Identity  979 bits Score | BCG_1244  WP_003898759.1  CAL71231.1  0.0 Expect value  100% Identity  979 bits Score | MAV_1762  WP_009976019.1  ABK65791.1  0.0 Expect value  59.6% Identity  601 bits Score | MAP_3763c  AAS06313.1  0.0 Expect value  54.5% Identity  525 bits Score | MSMEG_0409  WP_011726923.1  ABK73314.1  0.0 Expect value  54.8% Identity  577 bits Score |
| Rv1183  MmpL10  Probable conserved trans-membrane transport protein; PAT (penta-acyltrehalose) | BQ2027_MB1215  P65373.1  0.0 Expect value  100% Identity  2020 bits Score | BCG_1245  CAL71232.1  0.0 Expect value  100% Identity  2020 bits Score | MAV_1761  ABK67795.1  0.0 Expect value  60.1% Identity  1117 bits Score | MAP_2232  AAS04549.1  0.0 Expect value  57.1% Identity  923 bits Score | MSMEG_0410  WP_011726924.1  ABK75741.1  0.0 Expect value  57.1% Identity  1095 bits Score |
| Rv1184c  Chp2  Possible exported protein;  PAT (penta-acyltrehalose) | BQ2027_MB1216C  WP_003406195.1  0.0 Expect value  100% Identity  719 bits Score | BCG_1246c  WP_003406195.1  0.0 Expect value  100% Identity  719 bits Score | MAV_1760  WP_011724324.1  1e-100 Expect value  46.0% Identity  300 bits Score | MAP_2234  WP_010949528.1  1e-100 Expect value  46.0% Identity  300 bits Score | MSMEG_0412  WP_011726926.1  ABK73879.1  8e-96 Expect value  45.2% Identity  288 bits Score |
| Rv1185c  FadD21  Probable fatty-acid-AMP ligase;  DAT (diacyltrehalose) | BQ2027_MB1217C  WP_003406196.1  P63524.1  0.0 Expect value  100% Identity  1189 bits Score | BCG_1247c  WP_003406196.1  0.0 Expect value  100% Identity  1189 bits Score | MAV_1328  WP_009975522.1  ABK64909.1  0.0 Expect value  71.3% Identity  833 bits Score | MAP_2596  WP_003878484.1  0.0 Expect value  71.2% Identity  831 bits Score | MSMEG_0411  WP_011726925.1  ABK71017.1  0.0 Expect value  60.7% Identity  679 bits Score |
| Rv1196  PPE18  PPE family protein | BQ2027_MB1228  WP_010950503.1  0.0 Expect value  99.0% Identity  746 bits Score | BCG_1256  WP_011799164.1  0.0 Expect value  93.1% Identity  605 bits Score | MAV_2006  WP_011724528.1  ABK68797.1  6e-54 Expect value  41.1% Identity  182 bits Score | MAP_1515  WP_003877896.1  AAS03832.1  2e-48 Expect value  40.6% Identity  166 bits Score | MSMEG_0619  WP_011727070.1  ABK70912.1  5e-17 Expect value  41.2% Identity  80.9 bits Score |
| Rv1204c  Conserved hypothetical protein | BQ2027_MB1236C  WP_003406237.1  0.0 Expect value  100% Identity  1088 bits Score | BCG_1264c  WP_003406237.1  CAL71251.1  0.0 Expect value  100% Identity  1088 bits Score | MAV_1348  WP_011724081.1  ABK64429.1  3e-130 Expect value  53.1% Identity  394 bits Score | MAP_1875c  AAS04192.1  1e-68 Expect value  34.8% Identity  235 bits Score | MSMEG_5088  ABK71274.1  1e-120 Expect value  46.3% Identity  370 bits Score |
| Rv1252c  LprE  Probable lipoprotein | BQ2027_MB1284C  WP_003406334.1  P65313.1  1e-146 Expect value  100% Identity  403 bits Score | BCG_1312c  WP_003406334.1  1e-146 Expect value  100% Identity  403 bits Score | MAV_1400  ABK66416.1  2e-85 Expect value  69.2% Identity  248 bits Score | MAP_2522  WP_003875559.1  AAS04839.1  2e-86 Expect value  70.2% Identity  251 bits Score | MSMEG_5043  WP_003896449.1  ABK76075.1  3e-74 Expect value  62.9% Identity  220 bits Score |
| Rv1324  Possible thioredoxin | BQ2027_MB1359  WP_003406887.1  P64808.1  0.0 Expect value  100% Identity  595 bits Score | BCG_1386  WP_003406887.1  CAL71373.1  0.0 Expect value  100% Identity  595 bits Score | MAV_1545  WP_011724214.1  ABK66935.1  2e-148 Expect value  76.6% Identity  416 bits Score | MAP_2435c  WP_003873205.1  AAS04752.1  1e-148 Expect value  76.6% Identity  417 bits Score | MSMEG_4917  WP_011730190.1  ABK69619.1  3e-141 Expect value  68.1% Identity  399 bits Score |
| Rv1468c  PE_PGRS29  PE-PGRS family protein | BQ2027_MB3626C  SIU02253.1  1e-34 Expect value  69.1% Identity  130 bits Score | BCG_3660c  [CAL73649.1](https://www.ncbi.nlm.nih.gov/protein/CAL73649.1?report=genbank&log$=prottop&blast_rank=4&RID=6XF45VGU015)  9e-35 Expect value  69.1% Identity  130 bits Score | MAV_1346  [WP_010949655.1](https://www.ncbi.nlm.nih.gov/protein/WP_010949655.1?report=genbank&log$=protalign&blast_rank=3&RID=6XFD30MR016)  [ABK65871.1](https://www.ncbi.nlm.nih.gov/protein/ABK65871.1?report=genbank&log$=protalign&blast_rank=3&RID=6XFD30MR016)  3e-16 Expect value  51.1% Identity  75.5 bits Score | MAP_4144  [AAS06694.1](https://www.ncbi.nlm.nih.gov/protein/AAS06694.1?report=genbank&log$=prottop&blast_rank=1&RID=6XFTH28A016)  4e-35 Expect value  65.7% Identity  125 bits Score | No Gene Identified |
| Rv1479  MoxR1  Probable transcriptional regulatory protein | BQ2027_MB1515  WP_003407527.1  0.0 Expect value  100% Identity  755 bits Score | BCG_1541  WP_003407527.1  CAL71528.1  0.0 Expect value  100% Identity  755 bits Score | MAV_3299  WP_003876097.1  ABK66681.1  0.0 Expect value  92.6% Identity  636 bits Score | MAP_1205  WP_003876097.1  AAS03522.1  0.0 Expect value  92.6% Identity  636 bits Score | MSMEG_3147  ABK72180.1  0.0 Expect value  86.3% Identity  589 bits Score |
| Rv1635c  Probable mannosyltransferase conserved transmembrane protein  LAM (lipoarabinomannan) | BQ2027_MB1661C  WP_003408080.1  0.0 Expect value  100% Identity  1106 bits Score | BCG_1673c  WP_003408080.1  0.0 Expect value  100% Identity  1106 bits Score | MAV_3138  ABK67600.1  0.0 Expect value  70.1% Identity  675 bits Score | MAP_1338c  AAS03655.1  0.0 Expect value  68.1% Identity  703 bits Score | No Gene Identified |
| Rv1743  PknE  Probable transmembrane serine /threonine-protein kinase E | BQ2027_MB1772  WP_010950585.1  SIU00375.1  0.0 Expect value  99.8% Identity  1151 bits Score | BCG_1782  WP_003898998.1  0.0 Expect value  100% Identity  1154 bits Score | MAV_1417  WP_003875576.1  ABK67841.1  6e-139 Expect value  63.7% Identity  414 bits Score | MAP_2504  AAS04821.1  9e-141 Expect value  66.4% Identity  410 bits Score | MSMEG_4366  ABK71684.1  1e-98 Expect value  56.3% Identity  307 bits Score |
| Rv1759c  Wag22  PE-PGRS family protein | BQ2027_MB1789C  SIU00392.1  0.0 Expect value  99.9% Identity  1266 bits Score | BCG_1799c  CAL71786.1  0.0 Expect value  97.4% Identity  1212 bits Score | MAV_1346  WP_010949655.1  ABK65871.1  5e-16 Expect value  48.5% Identity  77 bits Score | MAP_4144  AAS06694.1  3e-31 Expect value  62.2% Identity  119 bits Score | No Gene Identified |
| Rv1821  SecA2  Possible preprotein translocase ATPase | BQ2027_MB1852  P66786.1  0.0 Expect value  100% Identity  1636 bits Score | BCG_1856  A1KJN3.1  0.0 Expect value  100% Identity  1636 bits Score | MAV_2894  WP_009977026.1  0.0 Expect value  90.6% Identity  1368 bits Score | MAP_1534  WP_003877905.1  0.0 Expect value  90.6% Identity  1396 bits Score | MSMEG_3654  WP_011729234.1  ABK70947.1  0.0 Expect value  82.6% Identity  1258 bits Score |
| Rv1831  Hypothetical protein | BQ2027_MB1862  P64900.1  1e-59 Expect value  100% Identity  174 bits Score | BCG_1866  CAL71853.1  1e-59 Expect value  100% Identity  174 bits Score | No Gene Identified | MAP_1544  AAS03861.1  2e-16 Expect value  55.7% Identity  65.1 bits Score | No Gene Identified |
| Rv1908c  KatG  Catalase-peroxidase-peroxynitritase T | BQ2027_MB1943C  WP_003901285.1  P46817.2  0.0 Expect value  99.9% Identity  1510 bits Score | BCG_1947c  WP_003901285.1  A1KJX3.1  0.0 Expect value  99.9% Identity  1510 bits Score | MAV_2753  WP_009976853.1  A0QGA4.1  0.0 Expect value  71.0% Identity  1007 bits Score | MAP_1668c  WP_003877970.1  AAS03985.1  0.0 Expect value  70.7% Identity  1001 bits Score | MSMEG_6384  WP_011731250.1  A0R609.1  0.0 Expect value  71.5% Identity  1055 bits Score |
| Rv2181  α (1->2) mannosyltransferase  LAM (lipoarabinomannan) | BQ2027_MB2203  WP_003411339.1  0.0 Expect value  100% Identity  824 bits Score | BCG_2196  WP_003411339.1  0.0 Expect value  100% Identity  824 bits Score | MAV_2312  ABK68984.1  0.0 Expect value  78.5% Identity  564 bits Score | MAP_1919  WP_010949412.1  0.0 Expect value  75.6% Identity  568 bits Score | MSMEG_4247  A0R036.1  6e-167 Expect value  63.2% Identity  474 bits Score |
| Rv2188c  PimB  Mannosyltransferase;  PIM (phosphatidyl-inositol mannoside) | BQ2027_MB2211C  WP_003411369.1  0.0 Expect value  100% Identity  760 bits Score | BCG_2204c  WP_003411369.1  0.0 Expect value  100% Identity  760 bits Score | MAV_2306  WP_011724716.1  ABK66338.1  0.0 Expect value  80.9% Identity  546 bits Score | MAP_1926c  WP_003872256.1  AAS04243.1  0.0 Expect value  81.2% Identity  550 bits Score | MSMEG_4253  A0R043.1  0.0 Expect value  71.7% Identity  546 bits Score |
| Rv2234  PtpA  Phosphotyrosine protein phosphatase | BQ2027_MB2258  WP_003411510.1  P65717.1  1e-120 Expect value  100% Identity  334 bits Score | BCG_2251  WP_003411510.1  9e-121 Expect value  100% Identity  334 bits Score | MAV_2206  WP_009976459.1  ABK65603.1  1e-99 Expect value  81.0% Identity  281 bits Score | MAP_1985  WP_003878184.1  8e-100 Expect value  81.6% Identity  281 bits Score | MSMEG_4309  WP_011729724.1  ABK75919.1  3e-78 Expect value  72.0% Identity  228 bits Score |
| Rv2416c  Eis  Enhanced intracellular survival protein | BQ2027_MB2439C  P59772.2  0.0 Expect value  99.7% Identity  803 bits Score | BCG_2432c  A1KLA8.2  0.0 Expect value  100% Identity  805 bits Score | MAV_1658  WP_011724278.1  ABK67236.1  1e-67 Expect value  35.1% Identity  218 bits Score | MAP_2325  WP_003873099.1  AAS04642.1  1e-67 Expect value  34.9% Identity  217 bits Score | MSMEG_3513  WP_011729122.1  ABK75645.1  6e-149 Expect value  57.6% Identity  426 bits Score |
| Rv2445c  NdkA  Probable nucleoside diphosphate kinase | BQ2027_MB2472C  WP_003412592.1  P84283.1  3e-96 Expect value  100% Identity  270 bits Score | BCG_2465c  WP_003412592.1  A1KLE1.1  3e-96 Expect value  100% Identity  270 bits Score | MAV_1727  WP_011724307.1  ABK66414.1  2e-82 Expect value  84.6% Identity  236 bits Score | MAP_2268c  WP_003875860.1  Q73XP1.1  6e-82 Expect value  83.8% Identity  234 bits Score | MSMEG_4627  WP_011729972.1  ABK70634.1  7e-79 Expect value  81.5% Identity  227 bits Score |
| Rv2610c  PimA  α-mannosyltransferase;  PIM (phosphatidyl-inositol mannoside) | BQ2027_MB2642C  WP_003413478.1  Q7TY88.1  0.0 Expect value  100% Identity  760 bits Score | BCG_2635c  WP_003413478.1  0.0 Expect value  100% Identity  760 bits Score | MAV_3486  WP_009977797.1  ABK67391.1  0.0 Expect value  88.1% Identity  639 bits Score | MAP_2712c  WP_003878556.1  0.0 Expect value  87.8% Identity  636 bits Score | MSMEG_2935  WP_011728703.1  ABK72422.1  0.0 Expect value  82.2% Identity  620 bits Score |
| Rv2741  PE_PGRS47  PE-PGRS family protein | BQ2027_MB3626C  SIU02253.1  5e-34 Expect value  66.9% Identity  131 bits Score | BCG_2756  CAL72744.1  7e-42 Expect value  100% Identity  152 bits Score | MAV_1346  ABK65871.1  4e-14 Expect value  50.5% Identity  70.5 bits Score | MAP_4144  AAS06694.1  3e-33 Expect value  63.6% Identity  123 bits Score | No Gene Identified |
| Rv2795c  Conserved hypothetical protein | BQ2027_MB2818C  WP_003414151.1  0.0 Expect value  100% Identity  674 bits Score | BCG_2813c  CAL72801.1  0.0 Expect value  100% Identity  674 bits Score | MAV_3684  WP_011725617.1  ABK69043.1  0.0 Expect value  88.4% Identity  599 bits Score | MAP_2900c  AAS05217.1  0.0 Expect value  88.7% Identity  599 bits Score | MSMEG_2647  WP_003894029.1  ABK69750.1  0.0 Expect value  85.2% Identity  572 bits Score |
| Rv2878c  mpt53  soluble secreted antigen | BQ2027_MB2903C  [SIU01524.1](https://www.ncbi.nlm.nih.gov/protein/1133642328)  2e-129 Expect value  100% Identity  347 bits Score | BCG_2900c  [CAL72889.1](https://www.ncbi.nlm.nih.gov/protein/121494408)  2e-129 Expect value  100% Identity  347 bits Score | MAV_3729  [ABK67260.1](https://www.ncbi.nlm.nih.gov/protein/ABK67260.1?report=genbank&log$=prottop&blast_rank=2&RID=6XHCY9CS014)  7e-91 Expect value  78.2% Identity  259 bits Score | MAP_2942c  [AAS05259.1](https://www.ncbi.nlm.nih.gov/protein/AAS05259.1?report=genbank&log$=prottop&blast_rank=1&RID=6XHHSE7R014)  6e-91 Expect value  72.5% Identity  260 bits Score | No Gene Identified |
| Rv2928  TesA  Probable thioesterase;  PDIM (phthiocerol dimycocerosate) | BQ2027_MB2953  WP_003414828.1  P63461.1  0.0 Expect value  100% Identity  540 bits Score | BCG_2950  WP_003414828.1  0.0 Expect value  100% Identity  540 bits Score | MAV_2010  ABK65888.1  3e-22 Expect value  29.7% Identity  89.4 bits Score | MAP_3745  WP_010950089.1  AAS06295.1  6e-26 Expect value  28.8% Identity  99.8 bits Score | MSMEG_4514  WP_003895881.1  ABK73080.1  8e-26 Expect value  31.4% Identity  100 bits Score |
| Rv2930  FadD26  Fatty-acid-AMP ligase; PDIM (phthiocerol dimycocerosate) | BQ2027_MB2955  WP_003904960.1  Q7TXM1.1  0.0 Expect value  99.8% Identity  1184 bits Score | BCG_2952  WP_003904960.1  0.0 Expect value  99.8% Identity  1184 bits Score | MAV_1328  WP_009975522.1  ABK64909.1  0.0 Expect value  56.9% Identity  630 bits Score | MAP_3752  WP_010950094.1  AAS06302.1  0.0 Expect value  58.5% Identity  681 bits Score | MSMEG_4731  WP_011730046.1  ABK71167.1  0.0 Expect value  54.2% Identity  623 bits Score |
| Rv3151  NuoG  Probable NADH dehydrogenase I | BQ2027_MB3175  WP_003899937.1  P59962.1  0.0 Expect value  99.7% Identity  1618 bits Score | BCG_3174  WP_003899937.1  0.0 Expect value  99.7% Identity  1618 bits Score | MAV_4039  ABK64902.1  0.0 Expect value  83.4% Identity  1352 bits Score | MAP_3207  WP_041160705.1  0.0 Expect value  82.9% Identity  1326 bits Score | MSMEG_2057  WP_011728121.1  ABK69848.1  0.0 Expect value  71.1% Identity  1100 bits Score |
| Rv3310  SapM  Acid phosphatase | BQ2027_MB3338  WP_003417249.1  0.0 Expect value  99.7% Identity  608 bits Score | BCG_3375  WP_003417249.1  0.0 Expect value  99.7% Identity  608 bits Score | MAV_4287  WP_011725986.1  ABK68959.1  1e-147 Expect value  75.6% Identity  413 bits Score | MAP_3432  AAS05982.1  5e-147 Expect value  75.2% Identity  412 bits Score | No Gene Identified |
| Rv3451  Cut3  Probable cutinase precursor;  TDM (trehalose dimycolate) | BQ2027_MB3481  WP_003418336.1  P0A537.1  0.0 Expect value  100% Identity  522 bits Score | BCG_3517  WP_011799351.1  CAL73506.1  0.0 Expect value  100% Identity  520 bits Score | MAV_4394  WP_009978946.1  ABK67856.1  6e-93 Expect value  63.0% Identity  273 bits Score | MAP_4237c  WP_003873439.1  AAS06787.1  1e-92 Expect value  63.0% Identity  272 bits Score | MSMEG_2095  WP_011728147.1  ABK75026.1  6e-92 Expect value  62.2% Identity  272 bits Score |
| Rv3484  CpsA  Possible conserved protein | BQ2027_MB3514  WP_003900866.1  0.0 Expect value  100% Identity  1038 bits Score | BCG_3548  WP_003900866.1  0.0 Expect value  100% Identity  1038 bits Score | MAV_0673  ABK67514.1  0.0 Expect value  76.0% Identity  771 bits Score | MAP_0579c  WP_010948897.1  AAS02896.1  0.0 Expect value  76.0% Identity  771 bits Score | MSMEG_0107  WP_011726672.1  ABK75496.1  2e-94 Expect value  41.0% Identity  295 bits Score |
| Rv3615c  EspC  ESX-1 secretion-associated protein | BQ2027_MB3645C  WP_003899599.1  P65088.1  1e-73 Expect value  100% Identity  210 bits Score | BCG_3679c  WP_003899599.1  1e-73 Expect value  100% Identity  210 bits Score | No Gene Identified | No Gene Identified | No Gene Identified |
| Rv3654c  Conserved hypothetical protein | BQ2027_MB3678C  SIU02306.1  4e-52 Expect value  100% Identity  154 bits Score | BCG_3712c  CAL73701.1  4e-52 Expect value  100% Identity  154 bits Score | MAV_0514  WP_009974714.1  ABK68304.1  4e-04 Expect value  71.9% Identity  33.9 bits Score | No Gene Identified | MSMEG_6164  WP_011731083.1  ABK73945.1  2e-11 Expect value  63.7% Identity  53.5 bits Score |
| Rv3655c  Conserved hypothetical protein | BQ2027_MB3679C  SIU02307.1  2e-63 Expect value  100% Identity  186 bits Score | BCG_3713c  CAL73702.1  2e-63 Expect value  100% Identity  186 bits Score | MAV_0512  ABK69482.1  9e-21 Expect value  70.1% Identity  77.8 bits Score | MAP_0420  AAS02737.1  7e-26 Expect value  69.0% Identity  90.5 bits Score | MSMEG_6165  ABK72239.1  6e-14 Expect value  63.0% Identity  63.2 bits Score |
| Rv3727  Possible oxidoreductase | BQ2027_MB3754  WP_010950931.1  0.0 Expect value  99.8% Identity  1227 bits Score | BCG_3787  WP_010950931.1  CAL73777.1  0.0 Expect value  99.8% Identity  1227 bits Score | MAV_4796  WP_011726256.1  ABK66226.1  5e-08 Expect value  25.8% Identity  53.5 bits Score | MAP_3849  WP_003879238.1  AAS06399.1  6e-08 Expect value  25.8% Identity  53.1 bits Score | No Gene Identified |
| Rv3763  LpqH  19 kDa lipoprotein antigen precursor | BQ2027_MB3789  WP_003420544.1  P0A5J1.1  3e-109 Expect value  100% Identity  305 bits Score | BCG_3822  WP_003420544.1  A0A0H3M9Z0.1  3e-109 Expect value  100% Identity  305 bits Score | MAV_0679  ABK66326.1  4e-39 Expect value  48.0% Identity  127 bits Score | MAP_2048  AAS04365.1  8e-05 Expect value  27.6% Identity  38.5 bits Score | MSMEG_6316  WP_003897736.1  ABK74869.1  2e-46 Expect value  49.7% Identity  147 bits Score |
| Rv3765c  TcrX  Probable two component transcriptional regulatory protein | BQ2027_MB3791C  WP_003420552.1  2e-171 Expect value  100% Identity  468 bits Score | BCG_3824c  WP_003420552.1  CAL73814.1  2e-171 Expect value  100% Identity  468 bits Score | MAV_0304  ABK68332.1  4e-153 Expect value  94.0% Identity  422 bits Score | MAP_0259  AAS02576.1  4e-153 Expect value  94.0% Identity  422 bits Score | MSMEG_4990  ABK72654.1  5e-140 Expect value  85.9% Identity  390 bits Score |
| Rv3820c  PapA2  Possible conserved polyketide synthase associated protein | BQ2027_MB3850C  WP_010950943.1  Q7TVL3.1  0.0 Expect value  99.8% Identity  969 bits Score | BCG_3882c  WP_010950943.1  A1KQF4.1  0.0 Expect value  99.8% Identity  969 bits Score | MAV_1762  WP_009976019.1  ABK65791.1  1e-158 Expect value  49.8% Identity  456 bits Score | MAP_1694  WP_010949317.1  AAS04011.1  4e-176 Expect value  54.9% Identity  501 bits Score | MSMEG_4728  WP_011730043.1  ABK73350.1  1e-171 Expect value  53.8% Identity  489 bits Score |
| Rv3823c  MmpL8  Conserved integral membrane transport protein;  SL-1 (sulfoglycolipid 1) | BQ2027_MB3853C  WP_003908351.1  Q7TVL0.1  0.0 Expect value  99.8% Identity  2163 bits Score | BCG_3886c  WP_003908351.1  A1KQF8.1  0.0 Expect value  99.8% Identity  2163 bits Score | MAV_1761  ABK67795.1  0.0 Expect value  54.6% Identity  1032 bits Score | MAP_2232  AAS04549.1  0.0 Expect value  52.3% Identity  829 bits Score | MSMEG_4741  ABK69902.1  0.0 Expect value  50.7% Identity  1025 bits Score |
| Rv3824c  PapA1  Conserved polyketide synthase associated protein | BQ2027_MB3854C  WP_003899711.1  Q7TVK9.1  0.0 Expect value  100% Identity  1056 bits Score | BCG_3887c  WP_003899711.1  A1KQF9.1  0.0 Expect value  100% Identity  1056 bits Score | MAV_1762  WP_009976019.1  ABK65791.1  0.0 Expect value  53.6% Identity  526 bits Score | MAP_3763c  AAS06313.1  3e-174 Expect value  55.1% Identity  498 bits Score | MSMEG_0409  WP_011726923.1  ABK73314.1  3e-174 Expect value  51.5% Identity  497 bits Score |
| Rv3846  SodA  Superoxide dismutase | BQ2027_MB3876  WP_010950947.1  Q7TVI9.1  6e-154 Expect value  99.5% Identity  422 bits Score | BCG_3909  WP_010950947.1  CAL73899.1  6e-154 Expect value  99.5% Identity  422 bits Score | MAV_0182  WP_003872294.1  1e-130 Expect value  82.6% Identity  363 bits Score | MAP_0187c  WP_010948769.1  AAS02504.1  3e-130 Expect value  82.1% Identity  362 bits Score | MSMEG_6427  WP_011731275.1  A0R652.1  1e-127 Expect value  81.2% Identity  356 bits Score |
| Rv3875  EsxA  6 kDa early secretory antigenic target | BQ2027_MB3905  WP_003399963.1  P0A565.2  9e-65 Expect value  100% Identity  187 bits Score | BCG_3511c  CAL73500.1  0.001 Expect value  34.6% Identity  32.7 bits Score | MAV_4388  ABK67596.1  0.002 Expect value  37.5% Identity  32.3 bits Score | MAP_4243  WP_003879501.1  AAS06793.1  0.011 Expect value  34.2% Identity  30.4 bits Score | MSMEG_0066  WP_003891394.1  ABK75559.1  1e-42 Expect value  71.6% Identity  132 bits Score |
| Rv3903c  CpnT  Hypothetical alanine and proline rich protein | BQ2027_MB3933C  WP_003899759.1  0.0 Expect value  100% Identity  1698 bits Score | BCG_3960c  WP_003899759.1  A0A0H3MAA9.1  0.0 Expect value  100% Identity  1698 bits Score | MAV_4644  WP_011726171.1  ABK66967.1  2e-177 Expect value  53.9% Identity  530 bits Score | MAP_3998c  WP_010950198.1  AAS06548.1  5e-178 Expect value  50.1% Identity  533 bits Score | MSMEG_1870  WP_011727967.1  ABK69844.1  2e-21 Expect value  24.5% Identity  98.6 bits Score |

^a^Altschul, S.F.; Gish, W.; Miller, W.; Myers, E.W.; Lipman, D.J. Basic local alignment search tool. *J Mol Biol* **1990**, *215*, 403-410, doi:10.1016/S0022-2836(05)80360-2.

^b^https://blast.ncbi.nlm.nih.gov/Blast.cgi?PROGRAM=blastp&PAGE_TYPE=BlastSearch&BLAST_SPEC=&LINK_LOC=blasttab&LAST_PAGE=blastn.

^c^Pearson, W.R. An introduction to sequence similarity ("homology") searching. *Curr Protoc Bioinformatics* **2013**, *Chapter 3*, 311-318, doi:10.1002/0471250953.bi0301s42.

^d^The Expect value (E-value) indicates the statistical significance of the hit as the likelihood the hit was found by chance. The default setting is 0.01 and results in the range of 1 and above should be considered putative false positives.

^e^The bit score is defined as the following: The value S′ is derived from the raw alignment score S in which the statistical properties of the scoring system used have been taken into account. By normalizing a raw score using the formula, S´ = (λ × S - lnK)/ ln2, a “bit score” S′ is attained, which has a standard set of units: K is a constant associated with the scoring matrix and is the Gumble distribution constant. Because bit scores have been normalized with respect to the scoring system, they can be used to compare alignment scores from different searches. Higher numbers correspond to higher similarity.
