# Supplementary material for: Cell death pathways in response to Mycobacterium tuberculosis and other mycobacterial infections
Source: Infect Immun. 2025 Sep 9;93(10):e00401-25. doi: 10.1128/iai.00401-25 (PMC12519786; doi:10.1128/iai.00401-25)
Supplement: Table S2 — CDD host factors. [file iai.00401-25-s0002.docx]

**Supplemental Table S2.** A National Center for Biotechnology Information (NCBI) Conserved Domain Database^a^ (CDD) search^b^ within a protein was conducted for each gene homolog identified in Table S1 that are virulence factors involved in cell death pathways. Each *Mycobacterium* is listed with specific taxonomy identification and GenBank numbers. The locus tag, name of domain hit, accession number, amino acid interval and Expect value^c^ are provided.

| ***M. tuberculosis* H37Rv**  **taxid:83332**  **GenBank**  **AL123456.3** | ***M. bovis* AF2122-97**  **taxid:233413**  **GenBank**  **LT708304.1** | ***M. bovis* BCG str. Pasteur 1173P2**  **taxid:410289**  **GenBank AM408590.1** | ***M. avium* 104**  **taxid:243243**  **GenBank**  **CP000479.1** | ***M. avium* subsp. *paratuberculosis* K-10**  **taxid:262316**  **GenBank**  **AE016958.1** | ***M. smegmatis* mc^2^155**  **taxid:246196**  **GenBank**  **CP000480.1** |
| --- | --- | --- | --- | --- | --- |
| Rv0009  PpiB  COG0652  13-182 interval  2.04e-69 Expect value | BQ2027_MB0009  PpiB  COG0652  13-182 interval  2.04e-69 Expect value | BCG_0009  PpiB  COG0652  13-182 interval  2.04e-69 Expect value | MAV_0013  PpiB  COG0652  7-176 interval  2.25e-69 Expect value | MAP_0011  PpiB  COG0652  13-182 interval  6.75e-68 Expect value | MSMEG_0024  PpiB  COG0652  1-175 interval  4.04e-72 Expect value |
| Rv0109  PE  pfam00934  4-94 interval  3.58e-19 Expect value | BQ2027_MB0113  PE  pfam00934  4-94 interval  1.36e-18 Expect value | BCG_0142  PE  pfam00934  4-94 interval  1.36e-18 Expect value | MAV_1346  PE  pfam00934  4-94 interval  2.41e-21 Expect value  PPE-SVP  pfam12484  192-276 interval  1.13e-07 Expect value | MAP_4144  PE  pfam00934  4-94 interval  1.60e-32 Expect value | No Gene Identified  (Table S1) |
| Rv0153c  Oca4  COG2365  5-276 interval  6.66e-70 Expect value | BQ2027_MB0158C  Oca4  COG2365  5-276 interval  5.84e-70 Expect value | BCG_0189c  Oca4  COG2365  5-276 interval  5.84e-70 Expect value | MAV_5145  Oca4  COG2365  7-276 interval  2.70e-67 Expect value | MAP_3568c  Oca4  COG2365  1-151 interval  3.18e-39 Expect value | MSMEG_0100  Oca4  COG2365  10-267 interval  5.34e-70 Expect value |
| Rv0198c  PepO  COG3590  6-662 interval  0.00e+00 Expect value | BQ2027_MB0204C  PepO  COG3590  6-662 interval  0.00e+00 Expect value | BCG_0235c  PepO  COG3590  6-662 interval  0.00e+00 Expect value | MAV_4977  PepO  COG3590  2-661 interval  0.00e+00 Expect value | No Gene Identified (Table S1) | MSMEG_0234  PepO  COG3590  5-665 interval  0.00e+00 Expect value |
| Rv0222  PRK08252  PRK08252  10-262 interval  1.28e-144 Expect value | BQ2027_MB1099C  PRK05862  PRK05862  1-257 interval  1.75e-177 Expect value | BCG_3445  CaiD  [COG1024](https://www.ncbi.nlm.nih.gov/Structure/cdd/cddsrv.cgi?ascbin=8&maxaln=10&seltype=2&uid=COG1024)  41-291 interval  2.78e-66 Expect value | MAV_4951  PRK08252  PRK08252  13-264 interval  6.19e-133 Expect value | MAP_3658  PRK08252  PRK08252  13-264 interval  4.54e-132 Expect value | MSMEG_2227  PRK08252  PRK08252  1-253 interval  1.69e-162 Expect value |
| Rv0256c  PPE  COG5651  4-376 interval  1.86e-63 Expect value  PPE-PPW  pfam18878  500-547 interval  4.17e-15 Expect value | BQ2027_MB0262C  PPE  COG5651  4-376 interval  1.86e-63 Expect value  PPE-PPW  pfam18878  500-547 interval  4.17e-15 Expect value | BCG_0294c  PPE  COG5651  4-376 interval  1.86e-63 Expect value  PPE-PPW  pfam18878  500-547 interval  4.17e-15 Expect value | MAV_1329  PPE  COG5651  7-384 interval  9.38e-38 Expect value | MAP_2595  PPE  COG5651  4-420 interval  3.02e-40 Expect value | MSMEG_0619  PPE  pfam00823  8-158 interval  9.76e-51 Expect value  PPE  COG5651  4-402 interval  1.85e-50 Expect value  PPE-PPW  pfam18878  458-504 interval  1.50e-13 Expect value |
| Rv0288  WXG100_ESAT6  TIGR03930  3-92 interval  7.70e-14 Expect value | BQ2027_MB0296  WXG100_ESAT6  TIGR03930  3-92 interval  7.70e-14 Expect value | BCG_0328  WXG100_ESAT6  TIGR03930  3-92 interval  7.70e-14 Expect value | MAV_4865  EsxA  COG4842  5-97 interval  4.80e-13 Expect value | MAP_3784  EsxA  COG4842  4-96 interval  5.46e-13 Expect value | MSMEG_0621  WXG100_ESAT6  TIGR03930  3-92 interval  5.26e-12 Expect value |
| Rv0297  PE  pfam00934  4-58 interval  3.87e-10 Expect value | BQ2027_MB0305  PE  pfam00934  4-58 interval  1.29e-10 Expect value | BCG_0337  PE  pfam00934  4-58 interval  1.29e-10 Expect value | MAV_1346  PE  pfam00934  4-94 interval  2.41e-21 Expect value  PPE-SVP  pfam12484  192-276 interval  1.13e-07 Expect value | MAP_4144  PE  pfam00934  4-94 interval  1.60e-32 Expect value | No Gene Identified (Table S1) |
| Rv0410c  PknG_TPR  pfam16918  409-748 interval  0.00e+00 Expect value  PknG_rubred  pfam16919  12-150 interval  3.71e-83 Expect value  STKc_PknB_like  cd14014  150-394 interval  3.76e-55 Expect value | BQ2027_MB0418C  PknG_TPR  pfam16918  409-748 interval  0.00e+00 Expect value  PknG_rubred  pfam16919  12-150 interval  3.71e-83 Expect value  STKc_PknB_like  cd14014  150-394 interval  3.76e-55 Expect value | BCG_0449c  PknG_TPR  pfam16918  409-748 interval  0.00e+00 Expect value  PknG_rubred  pfam16919  12-150 interval  3.71e-83 Expect value  STKc_PknB_like  cd14014  150-394 interval  3.76e-55 Expect value | MAV_4751  PknG_TPR  pfam16918  422-761 interval  0.00e+00 Expect value  PknG_rubred  pfam16919  20-163 interval  3.31e-77 Expect value  STKc_PknB_like  cd14014  163-405 interval  1.37e-53 Expect value | MAP_3893c  PknG_TPR  pfam16918  422-761 interval  0.00e+00 Expect value  PknG_rubred  pfam16919  20-163 interval  9.07e-77 Expect value  STKc_PknB_like  cd14014  163-405 interval  2.34e-55 Expect value | MSMEG_0786  PknG_TPR  pfam16918  419-758 interval  1.06e-167 Expect value  PknG_rubred  pfam16919  21-160 interval  1.30e-82 Expect value  STKc_PknB_like  cd14014  160-402 interval  3.15e-57 Expect value |
| Rv0432  SodC  COG2032  59-240 interval  4.76e-37 Expect value | BQ2027_MB0440  SodC  COG2032  59-240 interval  4.76e-37 Expect value | BCG_0471  SodC  COG2032  59-240 interval  4.76e-37 Expect value | MAV_4722  SodC  COG2032  1-227 interval  6.45e-39 Expect value | MAP_3921  SodC  COG2032  1-227 interval  5.37e-39 Expect value | MSMEG_0835  SodC  COG2032  66-236 interval  7.80e-39 Expect value |
| Rv0757  OmpR  COG0745  21-235 interval  7.30e-83 Expect value | BQ2027_MB0780  OmpR  COG0745  21-235 interval  7.30e-83 Expect value | BCG_0809  OmpR  COG0745  21-235 interval  7.30e-83 Expect value | MAV_0701  OmpR  COG0745  13-227 interval  1.84e-83 Expect value | MAP_0591  OmpR  COG0745  13-227 interval  3.92e-83 Expect value | MSMEG_5872  OmpR  COG0745  13-227 interval  6.50e-82 Expect value |
| Rv1001  PRK01388  PRK01388  1-402 interval  0.00e+00 Expect value | BQ2027_MB1028  PRK01388  PRK01388  1-402 interval  0.00e+00 Expect value | BCG_1058  PRK01388  PRK01388  1-402 interval  0.00e+00 Expect value | MAV_1125  PRK01388  PRK01388  1-402 interval  0.00e+00 Expect value | MAP_0942  PRK01388  PRK01388  1-400 interval  0.00e+00 Expect value | MSMEG_5448  PRK01388  PRK01388  1-402 interval  0.00e+00 Expect value |
| Rv1016c  Lpp-LpqN  pfam10738  56-224 interval  1.52e-79 Expect value | BQ2027_MB1044C  Lpp-LpqN  pfam10738  56-189 interval  1.04e-63 Expect value | BCG_1073c  Lpp-LpqN  pfam10738  56-189 interval  1.04e-63 Expect value | MAV_0054  Lpp-LpqN  pfam10738  104-272 interval  2.04e-83 Expect value | MAP_0047c  Lpp-LpqN  pfam10738  146-314 interval  3.14e-83 Expect value | MSMEG_5429  Lpp-LpqN  pfam10738  41-210 interval  2.85e-79 Expect value |
| Rv1068c  PE  pfam00934  4-94 interval  1.23e-27 Expect value | BQ2027_MB1097C  PE  pfam00934  4-94 interval  7.28e-29 Expect value | BCG_1126c  PE  pfam00934  4-94 interval  7.55e-29 Expect value | MAV_2923  PE  pfam00934  2-49 interval  3.51e-09 Expect value | MAP_4144  PE  pfam00934  4-94 interval  1.60e-32 Expect value | No Gene Identified (Table S1) |
| Rv1159  pimE  PRK13375  27-431 interval  0.00e+00 Expect value | BQ2027_MB1190  pimE  PRK13375  27-431 interval  0.00e+00 Expect value | BCG_1220  pimE  PRK13375  27-431 interval  0.00e+00 Expect value | MAV_1298  pimE  PRK13375  1-394 interval  0.00e+00 Expect value | MAP_2624c  pimE  PRK13375  28-437 interval  0.00e+00 Expect value | MSMEG_5149  pimE  PRK13375  1-377 interval  0.00e+00 Expect value |
| Rv1180  PksD  COG3321  4-478 interval  0.00e+00 Expect value | BQ2027_MB1213  No Specific Hits | BCG_1243  No Specific Hits | MAV_1321  No Specific Hits | MAP_2604c  PksD  COG3321  4-760 interval  0.00e+00 Expect value | MSMEG_4727  No Specific Hits |
| Rv1181  No Specific Hits | BQ2027_MB1213  No Specific Hits | BCG_1243  No Specific Hits | MAV_1321  No Specific Hits | MAP_3764c  No Specific Hits | MSMEG_4727  No Specific Hits |
| Rv1182  No Specfic Hits | BQ2027_MB1214  No Specfic Hits | BCG_1244  No Specfic Hits | MAV_1762  No Specfic Hits | MAP_3763c  No Specfic Hits | MSMEG_0409  No Specfic Hits |
| Rv1183  actII  TIGR00833  1-967 interval  0.00e+00 Expect value | BQ2027_MB1215  actII  TIGR00833  1-967 interval  0.00e+00 Expect value | BCG_1245  actII  TIGR00833  1-967 interval  0.00e+00 Expect value | MAV_1761  actII  TIGR00833  22-1000 interval  0.00e+00 Expect value | MAP_2232  No Specific Hits | MSMEG_0410  actII  TIGR00833  10-985 interval  0.00e+00 Expect value |
| Rv1184c  PE-PPE  pfam08237  79-316 interval  3.60e-78 Expect value | BQ2027_MB1216C  PE-PPE  pfam08237  79-316 interval  3.60e-78 Expect value | BCG_1246c  PE-PPE  pfam08237  79-316 interval  3.60e-78 Expect value | MAV_1760  No Specific Hits | MAP_2234  No Specific Hits | MSMEG_0412  No Specific Hits |
| Rv1185c  FAAL_FadD21  NF038337  1-578 interval  0.00e+00 Expect value | BQ2027_MB1217C  FAAL_FadD21  NF038337  1-578 interval  0.00e+00 Expect value | BCG_1247c  FAAL_FadD21  NF038337  1-578 interval  0.00e+00 Expect value | MAV_1328  No Specific Hits | MAP_2596  No Specific Hits | MSMEG_0411  PRK05850  PRK05850  1-569 interval  0.00e+00 Expect value |
| Rv1196  PPE  pfam00823  6-148 interval  7.90e-52 Expect value  PPE  COG5651  3-381 interval  1.14e-45 Expect value | BQ2027_MB1228  PPE  pfam00823  6-148 interval  1.26e-51 Expect value  PPE  COG5651  3-380 interval  1.40e-44 Expect value | BCG_1256  PPE  pfam00823  6-148 interval  2.85e-51 Expect value  PPE  COG5651  3-380 interval  1.20e-44 Expect value | MAV_2006  PPE  pfam00823  5-148 interval  3.85e-54 Expect value  PPE  COG5651  1-394 interval  9.88e-52 Expect value | MAP_1515  PPE  pfam00823  6-163 interval  2.34e-44 Expect value  PPE  COG5651  3-352 interval  4.42e-39 Expect value | MSMEG_0619  PPE  pfam00823  8-158 interval  9.76e-51 Expect value  PPE  COG5651  4-402 interval  1.85e-50 Expect value  PPE-PPW  pfam18878  458-504 interval  1.50e-13 Expect value |
| Rv1204c  AAA_16  pfam13191  10-134 interval  7.40e-05 Expect value | BQ2027_MB1236C  AAA_16  pfam13191  10-134 interval  7.40e-05 Expect value | BCG_1264c  AAA_16  pfam13191  10-134 interval  7.40e-05 Expect value | MAV_1348  No Specific Hits | MAP_1875c  CitB  COG2197  809-875 interval  1.78e-17 Expect value | MSMEG_5088  AAA_22  pfam13401  23-104 interval  2.52e-07 Expect value |
| Rv1252c  Lipoprotein_21  pfam14041  113-191 interval  2.63e-14 Expect value | BQ2027_MB1284C  Lipoprotein_21  pfam14041  113-191 interval  2.63e-14 Expect value | BCG_1312c  Lipoprotein_21  pfam14041  113-191 interval  2.63e-14 Expect value | MAV_1400  Lipoprotein_21  pfam14041  105-184 interval  4.13e-15 Expect value | MAP_2522  Lipoprotein_21  pfam14041  112-191 interval  6.95e-15 Expect value | MSMEG_5043  Lipoprotein_21  pfam14041  91-170 interval  1.60e-16 Expect value |
| Rv1324  ybbN  cd02956  51-147 interval  5.24e-36 Expect value | BQ2027_MB1359  ybbN  cd02956  51-147 interval  5.24e-36 Expect value | BCG_1386  ybbN  cd02956  51-147 interval  5.24e-36 Expect value | MAV_1545  ybbN  cd02956  50-146 interval  1.18e-35 Expect value | MAP_2435c  ybbN  cd02956  50-146 interval  8.28e-36 Expect value | MSMEG_4917  ybbN  cd02956  45-141 interval  1.57e-35 Expect value  TPR_20  pfam14561  208-296 interval  1.76e-19 Expect value  LapB  COG2956  156-257 interval  3.74e-05 Expect value |
| Rv1468c  PE  p[fam00934](https://www.ncbi.nlm.nih.gov/Structure/cdd/cddsrv.cgi?ascbin=8&maxaln=10&seltype=2&uid=pfam00934)  4-83 interval  5.81e-16 Expect value | BQ2027_MB3626C  PE  [pfam00934](https://www.ncbi.nlm.nih.gov/Structure/cdd/cddsrv.cgi?ascbin=8&maxaln=10&seltype=2&uid=pfam00934)  4-81 interval  1.81e-23 Expect value | BCG_3660c  PE  [pfam00934](https://www.ncbi.nlm.nih.gov/Structure/cdd/cddsrv.cgi?ascbin=8&maxaln=10&seltype=2&uid=pfam00934)  4-81 interval  1.81e-23 Expect value | MAV_1346  PE  [pfam00934](https://www.ncbi.nlm.nih.gov/Structure/cdd/cddsrv.cgi?ascbin=8&maxaln=10&seltype=2&uid=pfam00934)  4-94 interval  2.41e-21 Expect value  PPE-SVP  [pfam12484](https://www.ncbi.nlm.nih.gov/Structure/cdd/cddsrv.cgi?ascbin=8&maxaln=10&seltype=2&uid=pfam12484)  192-276 interval  1.13e-07 Expect value | MAP_4144  PE  [pfam00934](https://www.ncbi.nlm.nih.gov/Structure/cdd/cddsrv.cgi?ascbin=8&maxaln=10&seltype=2&uid=pfam00934)  4-94 interval  1.60e-32 Expect value | No Gene Identified (Table S1) |
| Rv1479  MoxR  COG0714  42-339 interval  8.36e-145 Expect value | BQ2027_MB1515  MoxR  COG0714  42-339 interval  8.36e-145 Expect value | BCG_1541  MoxR  COG0714  42-339 interval  8.36e-145 Expect value | MAV_3299  MoxR  COG0714  45-342 interval  1.93e-142 Expect value | MAP_1205  MoxR  COG0714  45-342 interval  1.93e-142 Expect value | MSMEG_3147  MoxR  COG0714  12-309 interval  3.41e-143 Expect value |
| Rv1635c  COG5305  COG5305  56-400 interval  1.17e-56 Expect value | BQ2027_MB1661C  COG5305  COG5305  56-400 interval  1.17e-56 Expect value | BCG_1673c  COG5305  COG5305  56-400 interval  1.17e-56 Expect value | MAV_3138  COG5305  COG5305  2-348 interval  5.29e-46 Expect value | MAP_1338c  COG5305  COG5305  113-466 interval  4.97e-52 Expect value | No Gene Identified (Table S1) |
| Rv1743  STKc_PknB_like  cd14014  15-267 interval  3.38e-105 Expect value  DsbG  COG1651  398-565 interval  8.14e-15 Expect value | BQ2027_MB1772  STKc_PknB_like  cd14014  15-267 interval  1.30e-105 Expect value  DsbG  COG1651  398-565 interval  8.22e-15 Expect value | BCG_1782  STKc_PknB_like  cd14014  15-267 interval  3.38e-105 Expect value  DsbG  COG1651  398-565 interval  8.14e-15 Expect value | MAV_1417  STKc_PknB_like  cd14014  15-269 interval  8.38e-112 Expect value  PknH_C  pfam14032  415-600 interval  4.60e-61 Expect value | MAP_2504  STKc_PknB_like  cd14014  15-269 interval  8.26e-112 Expect value | MSMEG_4366  STKc_PknB_like  cd14014  18-271 interval  4.02e-114 Expect value |
| Rv1759c  PE  pfam00934  4-82 interval  1.00e-12 Expect value | BQ2027_MB1789C  No Conserved Domains | BCG_1799c  No Conserved Domains | MAV_1346  PE  pfam00934  4-94 interval  2.41e-21 Expect value  PPE-SVP  pfam12484  192-276 interval  1.13e-07 Expect value | MAP_4144  PE  pfam00934  4-94 interval  1.60e-32 Expect value | No Gene Identified (Table S1) |
| Rv1821  SecA2_Mycobac  TIGR04221  42-808 interval  0.00e+00 Expect value | BQ2027_MB1852  SecA2_Mycobac  TIGR04221  42-808 interval  0.00e+00 Expect value | BCG_1856  SecA2_Mycobac  TIGR04221  42-808 interval  0.00e+00 Expect value | MAV_2894  SecA2_Mycobac  TIGR04221  12-777 interval  0.00e+00 Expect value | MAP_1534  SecA2_Mycobac  TIGR04221  12-777 interval  0.00e+00 Expect value | MSMEG_3654  SecA2_Mycobac  TIGR04221  12-784 interval  0.00e+00 Expect value |
| Rv1831  No Conserved Domains | BQ2027_MB1862  No Conserved Domains | BCG_1866  No Conserved Domains | No Gene Identified (Table S1) | MAP_1544  No Conserved Domains | No Gene Identified (Table S1) |
| Rv1908c  KatG  COG0376  15-740 interval  0.00e+00 Expect value | BQ2027_MB1943C  KatG  COG0376  15-740 interval  0.00e+00 Expect value | BCG_1947c  KatG  COG0376  15-740 interval  0.00e+00 Expect value | MAV_2753  KatG  COG0376  19-748 interval  0.00e+00 Expect value | MAP_1668c  KatG  COG0376  19-748 interval  0.00e+00 Expect value | MSMEG_6384  KatG  COG0376  19-739 interval  0.00e+00 Expect value |
| Rv2181  No Specific Hits | BQ2027_MB2203  No Specific Hits | BCG_2196  No Specific Hits | MAV_2312  No Specific Hits | MAP_1919  No Specific Hits | MSMEG_4247  No Specific Hits |
| Rv2188c  GT4_PimA-like  cd03801  3-379 interval  3.99e-67 Expect value | BQ2027_MB2211C  GT4_PimA-like  cd03801  3-379 interval  3.99e-67 Expect value | BCG_2204c  GT4_PimA-like  cd03801  3-379 interval  3.99e-67 Expect value | MAV_2306  GT4_PimA-like  cd03801  3-375 interval  8.21e-64 Expect value | MAP_1926c  GT4_PimA-like  cd03801  3-375 interval  2.61e-64 Expect value | MSMEG_4253  GT4_PimA-like  cd03801  2-373 interval  1.17e-66 Expect value |
| Rv2234  LMWPTP  cd16343  6-149 interval  1.50e-56 Expect value | BQ2027_MB2258  LMWPTP  cd16343  6-149 interval  1.50e-56 Expect value | BCG_2251  LMWPTP  cd16343  6-149 interval  1.50e-56 Expect value | MAV_2206  LMWPTP  cd16343  6-149 interval  1.01e-55 Expect value | MAP_1985  LMWPTP  cd16343  6-149 interval  8.17e-56 Expect value | MSMEG_4309  LMWPTP  cd16343  5-148 interval  1.22e-50 Expect value |
| Rv2416c  PRK01346  PRK01346  1-402 interval  2.25e-171 Expect value | BQ2027_MB2439C  PRK01346  PRK01346  8-408 interval  4.79e-170 Expect value | BCG_2432c  PRK01346  PRK01346  8-408 interval  3.22e-171 Expect value | MAV_1658  PRK01346  PRK01346  2-411 interval  1.19e-167 Expect value | MAP_2325  PRK01346  PRK01346  2-411 interval  9.92e-170 Expect value | MSMEG_3513  PRK01346  PRK01346  1-402 interval  9.35e-174 Expect value |
| Rv2445c  ndk  PRK00668  2-135 interval  8.85e-85 Expect value | BQ2027_MB2472C  ndk  PRK00668  2-135 interval  8.85e-85 Expect value | BCG_2465c  ndk  PRK00668  2-135 interval  8.85e-85 Expect value | MAV_1727  ndk  PRK00668  2-135 interval  1.33e-85 Expect value | MAP_2268c  ndk  PRK00668  2-135 interval  3.89e-85 Expect value | MSMEG_4627  ndk  PRK00668  2-136 interval  9.68e-85 Expect value |
| Rv2610c  GT4_PimA-like  cd03801  2-365 interval  2.20e-70 Expect value | BQ2027_MB2642C  GT4_PimA-like  cd03801  2-365 interval  2.20e-70 Expect value | BCG_2635c  GT4_PimA-like  cd03801  2-365 interval  2.20e-70 Expect value | MAV_3486  GT4_PimA-like  cd03801  2-361 interval  4.12e-75 Expect value | MAP_2712c  GT4_PimA-like  cd03801  2-361 interval  1.01e-74 Expect value | MSMEG_2935  GT4_PimA-like  cd03801  2-361 interval  4.11e-76 Expect value |
| Rv2741  PE  pfam00934  4-83 interval  3.99e-21 Expect value | BQ2027_MB3626C  PE  pfam00934  4-81 interval  1.81e-23 Expect value  PE  pfam00934  17-90 interval  8.19e-19 Expect value | BCG_2756  PE  pfam00934  2-68 interval  3.66e-16 Expect value | MAV_1346  PE  pfam00934  4-94 interval  2.41e-21 Expect value  PPE-SVP  pfam12484  192-276 interval  1.13e-07 Expect value | MAP_4144  PE  pfam00934  4-94 interval  1.60e-32 Expect value | No Gene Identified (Table S1) |
| Rv2795c  CpdA  COG1409  16-281 interval  1.96e-16 Expect value | BQ2027_MB2818C  CpdA  COG1409  16-281 interval  1.96e-16 Expect value | BCG_2813c  CpdA  COG1409  16-281 interval  1.96e-16 Expect value | MAV_3684  CpdA  COG1409  13-278 interval  2.83e-16 Expect value | MAP_2900c  CpdA  COG1409  21-286 interval  2.93e-16 Expect value | MSMEG_2647  CpdA  COG1409  14-278 interval  1.28e-16 Expect value |
| Rv2878c  TlpA_like_ScsD_MtbDsbE  [cd03011](https://www.ncbi.nlm.nih.gov/Structure/cdd/cddsrv.cgi?ascbin=8&maxaln=10&seltype=2&uid=cd03011)  26-258 interval  1.06e-79 Expect value | BQ2027_MB2903C  TlpA_like_ScsD_MtbDsbE  [cd03011](https://www.ncbi.nlm.nih.gov/Structure/cdd/cddsrv.cgi?ascbin=8&maxaln=10&seltype=2&uid=cd03011)  42-168 interval  5.00e-47 Expect value | BCG_2900c  TlpA_like_ScsD_MtbDsbE  [cd03011](https://www.ncbi.nlm.nih.gov/Structure/cdd/cddsrv.cgi?ascbin=8&maxaln=10&seltype=2&uid=cd03011)  42-168 interval  5.00e-47 Expect value | MAV_3729  TlpA_like_ScsD_MtbDsbE  [cd03011](https://www.ncbi.nlm.nih.gov/Structure/cdd/cddsrv.cgi?ascbin=8&maxaln=10&seltype=2&uid=cd03011)  27-153 interval  1.57e-45 Expect value | MAP_2942c  TlpA_like_ScsD_MtbDsbE  [cd03011](https://www.ncbi.nlm.nih.gov/Structure/cdd/cddsrv.cgi?ascbin=8&maxaln=10&seltype=2&uid=cd03011)  42-168 interval  4.83e-46 Expect value | No Gene Identified (Table S1) |
| Rv2928  GrsT  COG3208  26-258 interval  1.06e-79 Expect value | BQ2027_MB2953  GrsT  COG3208  26-258 interval  1.06e-79 Expect value | BCG_2950  GrsT  COG3208  26-258 interval  1.06e-79 Expect value | MAV_2010  GrsT  COG3208  1-219 interval  1.03e-62 Expect value | MAP_3745  GrsT  COG3208  13-245 interval  4.04e-81 Expect value | MSMEG_4514  GrsT  COG3208  19-246 interval  1.74e-73 Expect value |
| Rv2930  PRK05850  PRK05850  6-583 interval  0.00e+00 Expect value | BQ2027_MB2955  PRK05850  PRK05850  6-583 interval  0.00e+00 Expect value | BCG_2952  PRK05850  PRK05850  6-583 interval  0.00e+00 Expect value | MAV_1328  No Specific Hits | MAP_3752  PRK05850  PRK05850  4-578 interval  0.00e+00 Expect value | MSMEG_4731  PRK05850  PRK05850  4-574 interval  0.00e+00 Expect value |
| Rv3151  PRK07860  PRK07860  13-806 interval  0.00e+00 Expect value | BQ2027_MB3175  PRK07860  PRK07860  13-806 interval  0.00e+00 Expect value | BCG_3174  PRK07860  PRK07860  13-806 interval  0.00e+00 Expect value | MAV_4039  PRK07860  PRK07860  3-793 interval  0.00e+00 Expect value | MAP_3207  PRK07860  PRK07860  13-771 interval  0.00e+00 Expect value | MSMEG_2057  PRK07860  PRK07860  12-794 interval  1.00e+00 Expect value |
| Rv3310  No Specific Hits | BQ2027_MB3338  No Specific Hits | BCG_3375  No Specific Hits | MAV_4287  No Specific Hits | MAP_3432  No Specific Hits | No Gene Identified (Table S1) |
| Rv3451  Cutinase  pfam01083  44-226 interval  2.27e-64 Expect value | BQ2027_MB3481  Cutinase  pfam01083  44-226 interval  2.27e-64 Expect value | BCG_3517  Cutinase  pfam01083  44-226 interval  3.50e-64 Expect value | MAV_4394  No Specific Hits | MAP_4237c  No Specific Hits | MSMEG_2095  No Specific Hits |
| Rv3484  Cps2a  COG1316  73-371 interval  4.68e-78 Expect value  LytR_C  pfam13399  380-464 interval  1.96e-11 Expect value | BQ2027_MB3514  Cps2a  COG1316  73-371 interval  4.68e-78 Expect value  LytR_C  pfam13399  380-464 interval  1.96e-11 Expect value | BCG_3548  Cps2a  COG1316  73-371 interval  4.68e-78 Expect value  LytR_C  pfam13399  380-464 interval  1.96e-11 Expect value | MAV_0673  Cps2a  COG1316  74-372 interval  9.34e-76 Expect value  LytR_C  pfam13399  382-466 interval  6.01e-10 Expect value | MAP_0579c  Cps2a  COG1316  73-371 interval  1.44e-75 Expect value  LytR_C  pfam13399  381-465 interval  6.62e-10 Expect value | MSMEG_0107  Cps2a  COG1316  71-375 interval  3.70e-60 Expect value  LytR_C  pfam13399  386-468 interval  3.90e-12 Expect value |
| Rv3615c  T7SS_ESX_EspC  pfam10824  1-100 interval  2.66e-28 | BQ2027_MB3645C  T7SS_ESX_EspC  pfam10824  1-100 interval  2.66e-28 | BCG_3679c  T7SS_ESX_EspC  pfam10824  1-100 interval  2.66e-28 | No Gene Identified (Table S1) | No Gene Identified (Table S1) | No Gene Identified (Table S1) |
| Rv3654c  No Conserved Domains | BQ2027_MB3678C  No Conserved Domains | BCG_3712c  No Conserved Domains | MAV_0514  No Conserved Domains | No Gene Identified (Table S1) | MSMEG_6164  tadE_like_DECH  TIGR03816  1-106 interval  1.74e-12 Expect value |
| Rv3655c  No Specific Hits | BQ2027_MB3679C  No Specific Hits | BCG_3713c  No Specific Hits | MAV_0512  No Specific Hits | MAP_0420  No Specific Hits | MSMEG_6165  No Specific Hits |
| Rv3727  COG3349  COG3349  26-545 interval  4.01e-64 Expect value | BQ2027_MB3754  COG3349  COG3349  26-545 interval  3.46e-64 Expect value | BCG_3787  COG3349  COG3349  26-545 interval  3.46e-64 Expect value | MAV_4796  No Specific Hits | MAP_3849  No Specific Hits | No Gene Identified (Table S1) |
| Rv3763  Myco_19_kDa  pfam05481  43-158 interval  2.60e-36 Expect value | BQ2027_MB3789  Myco_19_kDa  pfam05481  43-158 interval  2.60e-36 Expect value | BCG_3822  Myco_19_kDa  pfam05481  43-158 interval  2.60e-36 Expect value | MAV_0679  Myco_19_kDa  pfam05481  28-147 interval  7.52e-27 Expect value | MAP_2048  Myco_19_kDa  pfam05481  50-179 interval  6.92e-11 Expect value | MSMEG_6316  Myco_19_kDa  pfam05481  67-175 interval  2.41e-29 Expect value |
| Rv3765c  OmpR  COG0745  10-222 interval  4.03e-74 Expect value | BQ2027_MB3791C  OmpR  COG0745  10-222 interval  4.03e-74 Expect value | BCG_3824c  OmpR  COG0745  10-222 interval  4.03e-74 Expect value | MAV_0304  OmpR  COG0745  23-234 interval  1.62e-74 Expect value | MAP_0259  OmpR  COG0745  23-234 interval  1.62e-74 Expect value | MSMEG_4990  OmpR  COG0745  25-237 interval  1.97e-74 Expect value |
| Rv3820c  No Specific Hits | BQ2027_MB3850C  No Specific Hits | BCG_3882c  No Specific Hits | MAV_1762  No Specific Hits | MAP_1694  No Specific Hits | MSMEG_4728  No Specific Hits |
| Rv3823c  actII  TIGR00833  41-1034 interval  0.00e+00 Expect value | BQ2027_MB3853C  actII  TIGR00833  41-1034 interval  0.00e+00 Expect value | BCG_3886c  actII  TIGR00833  41-1034 interval  0.00e+00 Expect value | MAV_1761  actII  TIGR00833  22-1000 interval  0.00e+00 Expect value | MAP_2232  No Specific Hits | MSMEG_4741  actII  TIGR00833  15-985 interval  0.00e+00 Expect value |
| Rv3824c  No Specific Hits | BQ2027_MB3854C  No Specific Hits | BCG_3887c  No Specific Hits | MAV_1762  No Specific Hits | MAP_3763c  No Specific Hits | MSMEG_0409  No Specific Hits  MSMEG_4728  No Specific Hits |
| Rv3846  SodA  COG0605  5-193 interval  1.92e-94 Expect value | BQ2027_MB3876  SodA  COG0605  5-193 interval  1.74e-94 Expect value | BCG_3909  SodA  COG0605  5-193 interval  1.74e-94 Expect value | MAV_0182  SodA  COG0605  5-193 interval  3.94e-96 Expect value | MAP_0187c  SodA  COG0605  5-193 interval  5.78e-96 Expect value | MSMEG_6427  SodA  COG0605  5-193 interval  4.95e-100 Expect value |
| Rv3875  WXG100_ESAT6  TIGR03930  5-87 interval  7.95e-13 Expect value | BQ2027_MB3905  WXG100_ESAT6  TIGR03930  5-87 interval  7.95e-13 Expect value | BCG_3511c  EsxA  COG4842  27-120 interval  3.99e-11 Expect value | MAV_4388  WXG100  pfam06013  1-84 interval  1.75e-10 Expect value | MAP_4243  WXG100  pfam06013  7-90 interval  1.36e-10 Expect value | MSMEG_0066  EsxA  COG4842  7-95 interval  5.74e-12 Expect value |
| Rv3903c  TNT  pfam14021  751-846 interval  3.37e-16 Expect value | BQ2027_MB3933C  TNT  pfam14021  751-846 interval  3.37e-16 Expect value | BCG_3960c  TNT  pfam14021  751-846 interval  3.37e-16 Expect value | MAV_4644  WXG100  pfam06013  7-92 interval  2.29e-03 Expect value | MAP_3998c  WXG100  pfam06014  7-92 interval  2.45e-03 Expect value  TNT  pfam14021 793-885  793-885 interval  8.52e-16 Expect value | MSMEG_1870  COG5412  COG5412  22-443 interval  1.22e-10 Expect value |

^a^This is a superset including NCBI-curated domains and external data imported from Pfam (Protein Families), Simple Modular Architecture Research Tool (SMART), Clusters of Orthologous Groups (COG), PRK (Protein K(c)lusters), and The Institute for Genomic Research's database of protein families (TIGRFAMs).

^b^Version 3.21 - 62456 position-specific scoring matrix (PSSMs) located at the following website: https://www.ncbi.nlm.nih.gov/Structure/cdd/wrpsb.cgi.

^c^The Expect value (E-value) indicates the statistical significance of the hit as the likelihood the hit was found by chance. The default setting is 0.01 and results in the range of 1 and above should be considered putative false positives.
